# Phase computations and phase models for discrete molecular oscillators

**DOI:** 10.1186/1687-4153-2012-6

**Published:** 2012-06-11

**Authors:** Onder Suvak, Alper Demir

**Affiliations:** 1 Department of Electrical and Electronics Engineering, College of Engineering, Koç University Rumeli Feneri Yolu 34450 Sariyer Istanbul, Turkey

**Keywords:** discrete molecular oscillators, oscillator phase, noise, phase noise, numerical methods, Monte Carlo methods, Stochastic Simulation Algorithm (SSA), isochrons, phase equations, phase computation schemes, phase models

## Abstract

**Background:**

Biochemical oscillators perform crucial functions in cells, e.g., they set up circadian clocks. The dynamical behavior of oscillators is best described and analyzed in terms of the scalar quantity, *phase*. A rigorous and useful definition for phase is based on the so-called *isochrons *of oscillators. Phase computation techniques for continuous oscillators that are based on isochrons have been used for characterizing the behavior of various types of oscillators under the influence of perturbations such as noise.

**Results:**

In this article, we extend the applicability of these phase computation methods to biochemical oscillators as discrete molecular systems, upon the information obtained from a continuous-state approximation of such oscillators. In particular, we describe techniques for computing the instantaneous phase of discrete, molecular oscillators for stochastic simulation algorithm generated sample paths. We comment on the accuracies and derive certain measures for assessing the feasibilities of the proposed phase computation methods. Phase computation experiments on the sample paths of well-known biological oscillators validate our analyses.

**Conclusions:**

The impact of noise that arises from the discrete and random nature of the mechanisms that make up molecular oscillators can be characterized based on the phase computation techniques proposed in this article. The concept of isochrons is the natural choice upon which the phase notion of oscillators can be founded. The isochron-theoretic phase computation methods that we propose can be applied to discrete molecular oscillators of any dimension, provided that the oscillatory behavior observed in discrete-state does not vanish in a continuous-state approximation. Analysis of the full versatility of phase noise phenomena in molecular oscillators will be possible if a proper phase model theory is developed, without resorting to such approximations.

## 1. Introduction

### 1.1 Oscillators in biological and electronic systems

Oscillatory behavior is encountered in many types of systems including electronic, optical, mechanical, biological, chemical, financial, social and climatological systems. Carefully designed oscillators are intentionally introduced into many engineered systems to provide essential functionality for system operation. In electronic systems, oscillators are used to generate clock signals that are needed in the synchronization of operations in digital circuits and sampled-data systems. The periodic signal generated by an electronic oscillator or monochromatic light from a laser is used as a carrier and for frequency translation of signals in wireless and optical communication systems. Oscillatory behavior in biological systems is seen in population dynamics models (prey-predator systems), in neural systems [1], in the motor system, and in circadian rhythms [2]. Intracellular and intercellular oscillators of various types perform crucial functions in biological systems. Due to their essentialness, and intricate and interesting dynamic behavior, biological oscillations have been a research focus for decades. Genetic oscillators that are responsible for setting up the circadian rhythms have received particular attention [3]. Circadian rhythms are crucial for the survival of many species, and there are many health problems associated with the disturbance of these clocks in humans [4,5]. For instance, working night shifts has been recently listed as a probable cause of cancer by the World Health Organization [6-8]. A milestone in synthetic biology is the work in [9] reporting on a genetic regulatory network called the repressilator, essentially a synthetic genetic oscillator.

Oscillators in electronic and telecommunication systems are adversely affected by the presence of undesired disturbances in the system. Various types of disturbances such as noise affect the spectral and timing properties of the ideally periodic signals generated by oscillators, resulting in power spreading in the spectrum and jitter and phase drift in the time domain [10]. Unlike other systems which contain an implicit or explicit time reference, autonomously oscillating systems respond to noise in a peculiar and somewhat nonintuitive manner. Understanding the behavior of oscillators used in electronic systems in the presence of disturbances and noise has been a preoccupation for researchers for many decades [11]. The behavior of biological oscillators under various types of disturbances has also been the focus of a good deal of research work in the second half of the 20th century [1,2,12,13].

### 1.2 Phase models for oscillators

The dynamical behavior of oscillators is best described and analyzed in terms of the scalar quantity, *phase*. Of the pertaining notions in the literature, the most straightforward phase definition is obtained when a planar oscillator is expressed in polar coordinates, with amplitude and polar angle as the state variables. The usefulness of the polar angle as phase does not generalize to higher dimensional oscillators. In the general case, it is our conviction that the most rigorous and precise definition of phase is the one that is based on the so-called *isochrons *(formed from in-phase points in the state-space) of an oscillator [1,2,14,15]. The notion of isochrons was first proposed by Winfree [2,14] in 1974. It was later revealed that isochrons are intimately related to the notion of asymptotic phase in the theory of differential equations [16,17]. The isochron theoretic phase of a free-running, noiseless oscillator is simply time itself. Such an unperturbed oscillator serves as a perfect time keeper if it is in the process of converging to a limit cycle, even when it has not yet settled to a periodic steady-state solution. Perturbations make the actual phase deviate from time, due to the degrading impact of disturbances on the time keeping ability.

Phase is a quantity that compactly describes the dynamical behavior of an oscillator. One is then interested in computing the phase of a perturbed oscillator. If this can be done in a semi or fully analytical manner for a practical oscillator, one can draw conclusions and obtain useful characterizations in assessing the time keeping performance. Indeed, we observe in the literature that, in various disciplines, researchers have derived *phase equations *that compactly describe the dynamics of weakly perturbed oscillators [1,11]. It appears that a phase equation for oscillators has first been derived by Malkin [18] in his work on the reduction of weakly perturbed oscillators to their phase models [1], and the same equation has been subsequently reinvented by various other researchers in several disciplines [2,11,19]. This phase equation has been used in mathematical biology to study circadian rhythms and coupled oscillators in the models of neurological systems [1,2,20], and in electronics for the analysis of phase noise and timing jitter in oscillators [11,21]. Phase equations have great utility in performing (semi) analytical phase computations. However, simpler and more accurate schemes for numerical phase computations have been recently proposed [15,22]. In some applications, merely a technique for computing the instantaneous phase of an oscillator for a given perturbation is needed. In this case, not only the machinery of phase equations is not necessary but also one can perform *more accurate *phase computations in a much simpler and straightforward manner.

### 1.3 Phase computations for discrete oscillators

We have proposed in [15] a numerical method for the computation of quadratic approximations for the isochrons of oscillators. In [22], we have reviewed the derivation of the first-order phase equation (which is based on the linear approximations for isochrons [1,2,20]), with a formulation based on the isochron-theoretic oscillator phase. On top of this, in [22] we have also made use of again the quadratic isochron approximations of [15] to derive a novel second-order phase equation that is more accurate than the first-order. However, the phase equations [22] and phase computation schemes [15] discussed above are founded on continuous oscillators described by differential equations. Therefore, these models and techniques do not directly apply to the analysis of molecular oscillators with discrete-space models. In this article, we present a methodology, enabling the application of these continuous phase models [22] and the phase computation schemes [15] on biological oscillators modeled in a discrete manner at the molecular level. Our preliminary results recently appeared in a workshop presentation [23]. This article details and expands on our contributions over this methodology.

We now summarize the workflow followed in the methodology and also give an outline of the article. Section 2 provides background information describing how the discrete model of the oscillator is transformed into a continuous, differential equation model through a limiting process based on the assumption that the concentration of molecular species in the model of the oscillator are large so that discrete effects are negligible [24-30]. It should be particularly noted that the reaction events in an SSA sample path (as generated by Gillespie's Stochastic Simulation Algorithm (SSA) [25]) are the most crucial ingredients in translating the continuous-state formalism on oscillator phase for use on molecular oscillators.

Section 3 actually describes our major contribution, i.e., how discrete-state oscillator phase computation is accomplished using the paradigms of phase equations and phase computation schemes. Using the phase modeling techniques mentioned above, a continuous phase model (depending on the model developed in Section 2) is constructed and discretized. The noise sources in this discretized phase model are represented as a cumulation of the events occurring in the discrete model of the oscillator. This two-way continuous-discrete transformation mechanism enables us to perform phase computations for discrete, molecular oscillators based on the continuous phase model theory [22]. Moreover, the fact that the noise sources in the phase computation are synthesized from the same events in the SSA sample path makes one-to-one comparisons with full SSA [25] based simulations possible. The phase model constructed as such from the continuous-limit model of the oscillator is accurate when a large number of molecules exist for every species. However, in many biological molecular oscillators, the number of molecules can be quite small. Large deviations from the continuous limit for such oscillators cause computations via continuous first-order phase models based on linear isochron approximations to become inaccurate. This was the observation that prompted our work on the quadratic (as opposed to linear) approximation theory and computational techniques for the isochrons of oscillators [15,22]. With phase computation schemes based on quadratic isochron approximations [15], deviations from the continuous-deterministic limit are much better captured and more accurate phase computations for discrete oscillators even with few molecules can be performed.

In Section 4, we provide a brief literature review of the approaches taken in the phase noise analysis of oscillators. Several seminal articles in the literature [11,31-36] are categorized according to three classification schemes in particular: the nature of the oscillator model used, the nature of the analysis method, and the phase definition adopted. We also classify in Section 4 the approach proposed in this article within the same framework.

Section 5 provides performance results for the proposed phase computation methods running on intricate molecular oscillators. The results are as expected, i.e., phase equations are quite accurate and fast for oscillators in a larger volume with big molecule numbers for the species, but they lose accuracy when a smaller volume is considered and noise effects become pronounced. Phase computation schemes are always very accurate, even in smaller volumes, but they are not as fast as the equations. Several crucial points in the theory underlying the methods are also emphasized in the discussion throughout this section. Section 6 concludes the article and suggests some future research directions.

The next three sections constitute the detailed explanation of the proposed methods. Sections 7 and 8 are expanded versions of Sections 2 and 3, respectively, with hints and references to derivations. Section 9 explains how and where molecular oscillator models can be obtained to test the proposed algorithms, which types of information are obtained from the models in preparation for oscillator phase analysis, numerical implementation details for the proposed phase computation methods, and in this section are also derived the computational complexities for these methods.

## 2 Modeling and simulation of discrete molecular oscillators

Biochemical models for molecular oscillators are generally specified as a set of molecular species participating in a number of reactions with predefined propensities. These models based on a stochastic chemical kinetics formalism capture the inherent stochastic and noisy behavior arising from the discrete and random nature of molecules and reactions. The (instantaneous) number of each molecular species, i.e., reactant, constitutes the state of the model. The time-dependent state probabilities for the system are described precisely with the Chemical Master Equation (CME) [28]. The generic form of the CME is as in

(1) $$\begin{array}{l} \frac{\text{d}\;\mathbb{P}(x,t)}{\text{d}t}=\\ \sum_{j=1}^{M}\left[a_{j}(x-s_{j}\right)\;\mathbb{P}\;(x-s_{j},t)-a_{j}(x)\;\mathbb{P}\;(x\text{,}\;t)] \end{array}$$

Above in (1), **x **represents the state of a molecular oscillator. The solution of this equation yields ℙ(x,t), i.e., the probability that the oscillator is visiting a certain state **x **at time *t*. Also, in (1), *a*_*j*_(**x**) is called the *propensity *of the *j *th reaction (note that we have *M *possible reactions), while the oscillator is again visiting the state **x**. This propensity function facilitates the quantification of how much of a probability we have of reaction *j *occuring in the next infinitesimal time. The constant vector **s**_*j *_defines the changes in the numbers of molecules for the species constituting the oscillatory system, when reaction *j *occurs. The CME corresponds to a continuous-time Markov chain. Due to the exponential number of state configurations for the system, CME is generally very hard to construct and solve. Therefore, one prefers to generate sample paths for the system using Gillespie's SSA [25], whose ensemble obeys the probability law dictated by the CME.

Continuous state-space models for molecular oscillators that serve as approximations to the discrete model described above are also used. Based on the CME and employing certain assumptions and approximations, one may derive a continuous state-space model as a system of stochastic differential equations, known as the Chemical Langevin Equations (CLEs). A CLE is of the generic form in

(2) $$\frac{\text{d}\;X\left(t\right)}{\text{d}t}=S\;a\left(X\text{(}t\text{)}\right)+SD\left(\left[\sqrt{\text{a}\left(X\left(t\right)\right)}\right]\right)\xi \left(t\right)$$

Above in (2), **X**(*t*) is the state of the oscillator, i.e., the solution of the SDE for a particular realization. Vectors **s**_*j *_defined above are stacked side by side for all of the *M *reactions to compose the stoichiometric matrix **S **in (2). Note also that $D\left(\left[\sqrt{a\text{(}X\text{(}t\text{))}}\right]\right)$ is a square diagonal matrix with its diagonal entries given by $\sqrt{a_{j}\left(X\left(t\right)\right)}$ for *j *= 1, ..., *M*, with **a**(**X**(*t*)) the vector of propensity functions. The vector *ξ*(*t*) is composed of independent zero-mean Gaussian random variables with variance one. The deterministic limit of the CLEs is in turn called the Reaction Rate Equations (RREs). The generic form of an RRE is as in

(3) $$\frac{\text{d}\;X(t)}{\text{d}t}=\sum_{j=1}^{M}{\text{s}}_{j}\;a_{j}\text{(}X\text{(}t\text{))}=S\;a\text{(}X\text{(}t\text{))}$$

which is mathematically obtained by crossing out the second term on the right-hand side of (2). The RRE model for an oscillator has a solution that is perfectly periodic without noisy fluctuations. On the other hand, the solution of the CLEs produces oscillatory sample paths with fluctuations around the periodic orbit on top of the deterministic solution of the RREs [28].

**Figure 1:**
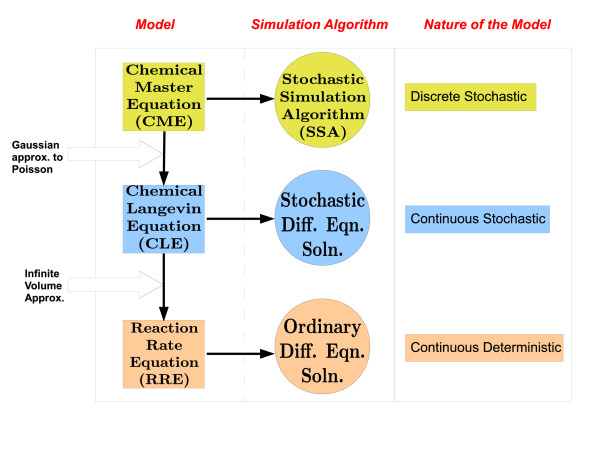
**Summary of molecular models and corresponding algorithms**. The models, their natures, and the simulation algorithms for these models are given. CME (of discrete stochastic nature) dictates the probability evolution of the ensemble of the sample paths generated by the SSA algorithm. Applying to the CME the *τ*-leap criterion and Gaussian approximation to Poisson random variables, CLE (continuous stochastic) is derived. CLE sample paths are obtained via appropriate SDE Solutions. The infinite volume approximation acting on the CLE leads us to the RRE (continuous deterministic), whose solutions we get through algorithms for ODEs.

The reader is referred to Figure 1, in which a summary of the models (along with their respective natures) for molecular oscillators and the algorithms used to solve these models are provided. The instantaneous phase computations we describe in this article are performed on the sample paths generated by SSA simulations based on a fully discrete model of the oscillator. However, the isochron characterization (computation of linear and quadratic isochron approximations) for the oscillator is based on the continuous-space RRE and CLE model, as we describe in the next section.

## 3 Phase computations based on Langevin models

**Figure 2:**
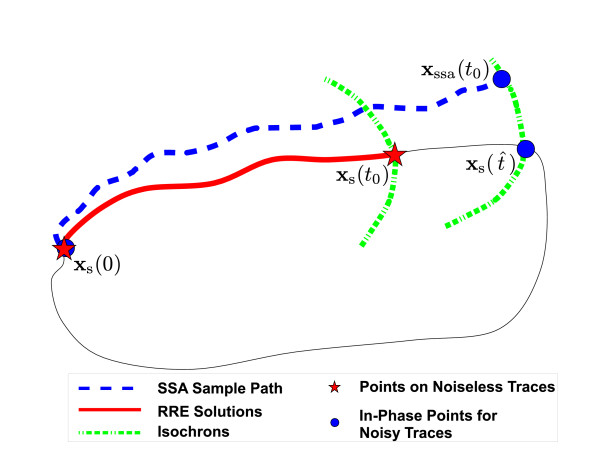
**Phase computation problem for oscillators**. The two trajectories **x**_s_(*t*), the periodic solution of the RRE, and **x**_ssa_(*t*), a sample path, start at the same point on the limit cycle, but at *t *= *t*_0 _they end up at different points and possibly on different isochrons. The point **x**_ssa_(*t*_0_) has registered a phase shift with respect to **x**_s_(*t*_0_). According to isochron theory, there is a point $x_{\text{s}}(\hat{t})$ that is on the same isochron as **x**_ssa_(*t*_0_), therefore the two points are in-phase. The time argument $\hat{t}$ of the point $x_{\text{s}}(\hat{t})$ is the instantaneous phase value of **x**_ssa_(*t*_0_). Phase computation methods aim to calculate this value $\hat{t}$.

In performing phase characterizations, we compute sample paths for the instantaneous phase $\hat{t}$ (in units of time) of a molecular oscillator. In the absence of noise and disturbances, i.e., for an unperturbed oscillator, the phase $\hat{t}$ is always exactly equal to time *t *itself, even if the oscillator is not at periodic steady-state. Perturbations and noise result in deviations in the phase $\hat{t}$ and cause it to be different from time *t *[1,2,11,15,22]. The perpetual effect of noise and disturbances causes this deviation in the phase $\hat{t}$ to accumulate. Our goal is to compute the instantaneous phase $\hat{t}$ that corresponds to an SSA generated sample path for a molecular oscillator. A pictorial description of this phase computation problem for oscillators is given in Figure 2.

**Figure 3:**
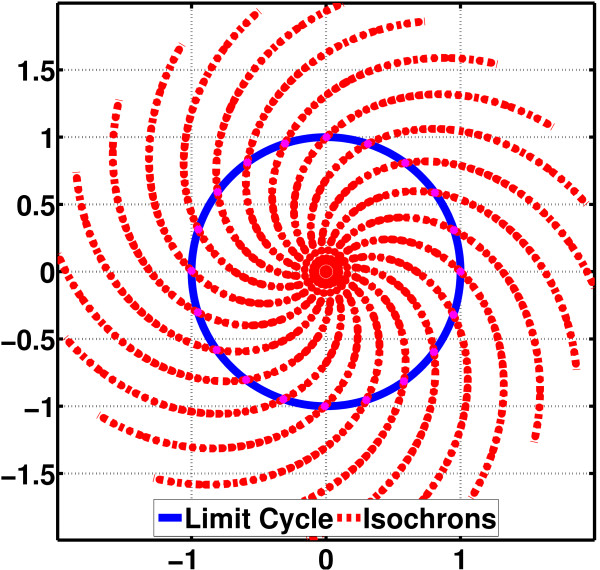
**Limit cycle and isochrons of a polar oscillator (figure from **[15]). For this oscillator, the isochrons are analytically calculable. Note that each isochron crosses the limit cycle exactly at a single point.

We assume that the deterministic RREs for a molecular oscillator have a stable periodic solution **x**_s_(*t*) that represents a periodic orbit or limit cycle. An isochron of an oscillator associated with the limit cycle **x**_s_(*t*) is a set of points (in the state-space) that have the same phase. For an oscillator with *N *state variables, each isochron is an *N *- 1-*dimensional hypersurface*. The union of isochrons covers the neighborhood of its periodic orbit [1,14]. See Figure 3 for the limit cycle and isochrons of a simple polar oscillator. Isochrons form the basis for a rigorous phase definition and phase computations for oscillators [22]. Another crucial quantity in devising phase computation schemes, in addition to isochrons, is the orbital deviation, i.e., the instantaneous difference between the noisy oscillator state and the in-phase point on the limit cycle (by definition, the two points are on the same isochron) [22].

The perturbation projection vector (PPV) **v**(*t*) is defined as the *gradient *of the *phase *$\hat{t}$ of an oscillator [22] on the limit cycle represented by **x**_s_(*t*). The PPV, which is equivalent to the infinitesimal *phase response curves (PRCs) *[1], is instrumental in forming linear approximations for the isochrons of an oscillator. The matrix **H**(*t*) is defined as the *Hessian *of the phase $\hat{t}$ (and the Jacobian of the PPV) [22] on the limit cycle. The phase Hessian **H**(*t*) is useful in forming quadratic approximations for the isochrons of an oscillator. The PPV **v**(*t*) and the Hessian **H**(*t*) can be computed using the techniques described in [15].

**Figure 4:**
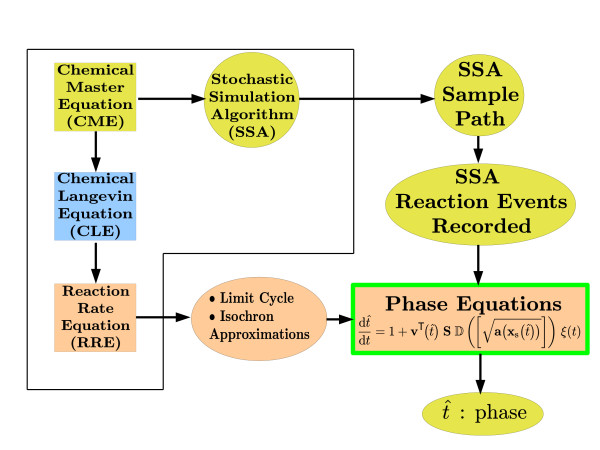
**Phase computations through phase equations methodology**. The events in the SSA-generated sample path are recorded. From the RRE, the limit cycle and isochron approximation information is computed. Phase equations make use of these two pieces of information to compute the instantaneous phase corresponding to each point in the sample path. The first-order phase equation as adapted to this methodology is given in the figure.

Phase equations (differential equations for the phase $\hat{t}$) can be derived based on the CLE model of an oscillator. Phase equations come in various flavors, depending on whether a linear or quadratic approximation is used for the isochrons and the orbital deviation [22]. The acclaimed phase equation, used in multiple disciplines [1,2,11], of the form

(4) $$\frac{\text{d}\;\hat{t}}{\text{d}t}=1+v^{\text{T}}(\hat{t})b(x_{\text{s}}(\hat{t}),t)$$

is based on linear isochron approximations and a linear differential equation for the orbital deviation (not shown here). Above, **b **is the noise excitation which is synthesized as a cumulation of the events that occur in the discrete, molecular level model of the oscillator. We call the model of (4) PhEqnLL (the first L for the isochron and the second one for the orbital deviation approximation, the natures of both of which are linear). We also have PhEqnQQ (quadratic approximations for both isochrons and orbital deviation) and PhEqnQL (quadratic approximations for isochrons and linear approximations for orbital deviation) [22]. See Figure 4 for a high-level representation of the phase computations methodology using phase equations.

**Figure 5:**
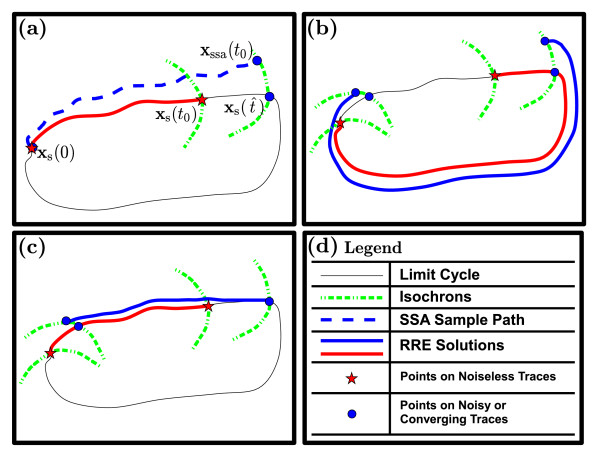
**Brute-force phase computation scheme (PhCompBF)**. (**a**) An SSA sample path and a noiseless RRE solution (running in parallel) end up at different isochrons at *t*_0_. (**b**) The last timepoints in the two simulations for part (**a**) are separately fed as initial conditions to the RRE in order to generate the two separate traces shown in this subfigure. The RRE solution that was already on the periodic orbit continues tracing it, and the off-orbit solution in turn approaches the limit cycle. (**c**) The off-orbit solution finally becomes periodic and the phase shift between the two RRE solutions can be found, switching to the plots in the time domain and applying appropriate algorithms to compute the phase shift. (**d**) Legend for the traces in the subplots.

**Figure 6:**
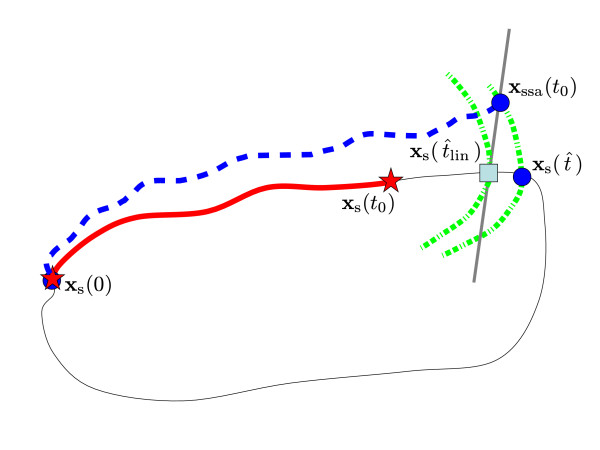
**Phase computation scheme depending on linear isochron approximations**. An isochron whose linear approximation passes through the point **x**_ssa_(*t*_0_) is found. The point where this hyperplane crosses the limit cycle is $x_{\text{s}}\left({\hat{t}}_{\text{lin}}\right)$, with ${\hat{t}}_{\text{lin}}$ the solution of this phase computation scheme. The difference between the exact solution $\hat{t}$ and the approximate solution ${\hat{t}}_{\text{lin}}$ is reduced if the isochrons are close to being linear.

**Figure 7:**
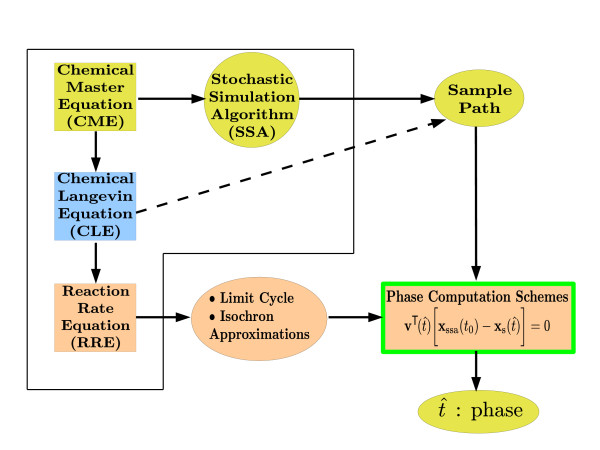
**Phase computation schemes methodology**. An SSA-generated sample path (alternatively one that is generated through the CLE) and the limit cycle and isochron approximation information are fed to the phase computation schemes, which compute the instantaneous phase corresponding to each point in the sample path. The algebraic equation for the scheme depending on linear isochron approximations is given as an example.

With the phase equations based on linear and quadratic isochron approximations, we can compute the phase of an oscillator without having to run SSA simulations based on its discrete, molecular model (unless a one-to-one comparison between the results of phase computations based on phase equations and SSA simulations is required). On the other hand, more accurate phase computations can be attained if they are based on, i.e., use information, from SSA simulations. In this scheme, we run an SSA simulation based on the discrete, molecular model of the oscillator. For points (in the state-space) on the sample path generated by the SSA simulation, we compute a corresponding phase by essentially determining the isochron on which the point in question lies. Here, one can either employ no approximations (PhCompBF) for the isochrons or perform phase computations based on linear (PhCompLin) or quadratic (PhCompQuad) isochron approximations. Brute-force phase computations without isochron approximations (PhCompBF) are computationally costly [15,22]. See Figure 5 for a pictorial description of PhCompBF. Phase computations based on isochron approximations and SSA simulations proceeds as follows: Let **x**_ssa_(*t*) be the sample path for the state vector of the oscillator that is being computed with SSA. We solve

(5) $$v^{⊺}(\hat{t})\left[x_{\text{ssa}}(t)-x_{\text{s}}(\hat{t})\right]=0$$

based on linear isochron approximations (PhCompLin)--or a similar equation that also involves the phase Hessian **H**(*t*) based on quadratic isochron approximations (PhCompQuad)--for the phase $\hat{t}$ that corresponds to **x**_ssa_(*t*). Figure 6 provides a description for PhCompLin. The above computation needs to be repeated for every time point *t *of interest. Above, for **x**_ssa_(*t*), we essentially determine the isochron (in fact, a linear or quadratic approximation for it) that passes through both the point $x_{\text{s}}(\hat{t})$ on the limit cycle and **x**_ssa_(*t*). The phase of $x_{\text{s}}(\hat{t})$, i.e., $\hat{t}$, is then the phase of **x**_ssa_(*t*) as well since they reside on the same isochron. We should note here that, even though **x**_ssa_(*t*) above is computed with an SSA simulation based on the discrete model of the oscillator, the steady-state periodic solution $x_{\text{s}}(\hat{t})$, the phase gradient $v\left(\hat{t}\right)$ and the Hessian $H\left(\hat{t}\right)$ (i.e., all of the information that is used in constructing the isochron approximations) are computed based on the continuous, RRE model of the oscillator. See Figure 7 for the high-level representation of the phase computations methodology using phase computation schemes. The phase computation schemes we describe here can be regarded as *hybrid *techniques that are based both on the continuous, RRE and also the discrete, molecular model of the oscillator. On the other hand, the phase computations based on phase equations are completely founded upon the continuous, RRE and CLE models of the oscillator.

In summary, we point out the acronyms and some properties of the proposed phase computation methods for convenience. The phase equations are PhEqnLL, PhEqnQL, and PhEqnQQ. The phase computation schemes are PhCompBF (the most accurate but computationally expensive method), PhCompLin, and PhCompQuad. The schemes employ no approximations in orbital deviation, therefore they are expected to be more accurate with respect to the equations. The equations, on the other hand, have low computational complexity and can generate results very fast. We also show in this article that there is a trade-off between accuracy and computational complexity for these methods.

## 4 Related work

A classification scheme for categorizing previous work, pertaining to the phase noise analysis of biochemical oscillators, can be described as follows.

First, we note that there are basically two types of models for inherently noisy biochemical oscillators, i.e., discrete and continuous-state. CME describes the probabilistic evolution of the states of an oscillator, and it is referred to as the most accurate characterization for discrete molecular oscillators. Through approximations, one derives from CME the CLE, a continuous-state noisy model. CLE can be used to extract crucial information about the continuous-state system that is an approximate representation of its discrete-state ancestor. We note here that, in oscillator phase noise analyses, mostly the continuous-state model has been utilized [11,31-36].

Second, the nature of the phase noise analyses conducted can be considered in two categories, i.e., semi-analytical techniques and sample path-based approaches. Semi-analytical techniques have been developed, in particular, for the stochastic characterization of phase diffusion in oscillators [11,31-36]. In biology, CLE has been used as a tool in illustrating and quantifying the phase diffusion phenomena [31-34,36]. Characterization and computations pertaining to phase diffusion in electronic oscillators were carried out through a stochastic phase equation and the probabilistic evolution of its solutions [11], noting that the phase equation used was derived from an SDE (a Stochastic Differential Equation describing a noisy electronic oscillator) that corresponds to the CLE for biochemical oscillators. In all, these semi-analytical techniques are based on the continuous-state model of an oscillator. Regarding sample path-based approaches, one may recall that, in discrete state, SSA is used to generate sample paths, whose ensemble obeys the CME. In continuous state, CLE can in turn be used to generate sample paths. A recent study [35] illustrates derivations of the crucial findings presented in [11,33,34] and adopts an approach for phase diffusion constant computation, based on the transient phase computation of CLE-generated sample paths in an ensemble.

Third, oscillator phase can be defined via two different methods. There are the Hilbert transform-based and the isochron-based definitions. The phase computation based on the Hilbert transform [37] takes the evolution of a single state variable within a sample path to compute the phases of all time points in the whole sample path. The Hilbert transform-based phase computation technique can be used to compute the phase of any oscillatory waveform, without any information as to where this waveform came from. The oscillatory waveform could belong to one of the state variables of an oscillator generated with a simulation. This method has been utilized in [31,35] for phase computations of sample paths. The isochron-theoretic phase (recall that an isochron portrait belongs to a limit cycle of the deterministic RRE) makes use of all of the state variables and equations for an oscillator. The isochron-based phase definition assigns a phase value to the points in the state space of the oscillator, making phase a property of the whole oscillator, not a property of just a certain state variable or a waveform obtained with a simulation of the oscillator [15,22]. Note that even though there appears to be empirical evidence [31,35] that there is a correspondence between the Hilbert transform-based and isochron-based phase definitions, a precise connection has not been worked out in the literature.

The hybrid phase computation techniques proposed in this article apply to discrete-state models and particularly the SSA generated sample paths of these models, based on the isochron-theoretic oscillator phase definition. Our approach is hybrid because isochrons are obtained based on the continuous model but the phase traces are computed for the sample paths generated by an SSA simulation that is based on the discrete model for an oscillator. This hybrid approach targets moderately noisy oscillators, within a container of not too large or small volume, consequently with not too high or low molecule numbers for the species in the system, respectively.

## 5 Results and discussion

We now present results obtained with the proposed methods for oscillator phase computations on several intricate molecular oscillators. Accuracy demonstrations and computational speed-up figures will be given with respect to PhCompBF, the brute-force scheme, which we accept as the golden reference for oscillator phase computations, since this method does not employ any approximations in either isochrons or orbital deviations. Section 5.1 below, in which we analyze the brusselator, contains details pertaining to the general flow of the phase computations and the preparatory procedures for all the methods. Sections 5.2 and 5.3 are brief sections illustrating the performance of the methods for oscillators called the oregonator and the repressilator, respectively. All simulations were run on a computer with an Intel i7 processor at 3.07 GHz and accommodating 6 GB of memory.

### 5.1 Brusselator

The Brusselator is a theoretical model for a type of autocatalytic reaction. The Brusselator actually describes a type of chemical clock, and the Belousov-Zhabotinsky (BZ in short) reaction is a typical example [38]. The model below in (6) has been largely adapted from [39], which is based on [38].

(6) $$\begin{array}{llll} \text{A} & \overset{k_{1}}{\to }\text{X}\; & & \;\\ \text{B + X} & \overset{k_{2}}{\to }\text{R + Y}\; & & \;\\ \text{Y + 2X} & \overset{k_{3}}{\to }3\text{X}\; & & \;\\ \text{X} & \overset{k_{4}}{\to }\text{S}\; & & \;\\ & \; & \end{array}$$

Parameter values in (6) are: *k*_1 _= 0.025 s^-1^, *k*_2 _= 1 s^-1 ^mL, *k*_3 _= 1 s^-1 ^(mL)^2^, and *k*_4 _= 0.01 s^-1^. Volume is set to 250 mL. Molecule numbers of A, B, R, and S are held, constant.

Several models and quantities must be derived from the reactions in (6) before moving onto phase analysis. The stoichiometric matrix in this case reads

(7) $$S=\left[\begin{array}{cccc} 1 & -1 & 1 & -1\\ 0 & 1 & -1 & 0 \end{array}\right]$$

where the first row is for the species X and the second is for Y. The columns each denote the changes in molecule numbers as a reaction takes place, e.g., column one is for the first reaction in (6). Let us also call *X *the random process denoting the instantaneous molecule number for the species X, similarly *Y *is for Y in the same fashion. Then, the random process vector **X **= [*X Y*]^Τ ^concatenates these numbers for convenience. The propensity functions for the reactions can be written as

(8) $$\begin{array}{llll} a_{1}\left(X\right) & =k_{1}\;A\; & & \;\\ a_{2}\left(X\right) & =\frac{k_{2}B\;X}{\Omega }\; & & \;\\ a_{3}\left(X\right) & =\frac{k_{3}Y\;X\left(X-1\right)}{\Omega^{2}}\; & & \;\\ a_{4}\left(X\right) & =k_{4}\;X\; & & \;\\ & \; & \end{array}$$

where Ω denotes the volume parameter. Using (8), the CME for the Brusselator can be derived in line with (1) as

(9) $$\begin{array}{llll} \frac{\text{d}\;\mathbb{P}\left(X,Y;t\right)}{\text{d}t}= & -\left[k_{1}A+\frac{k_{2}B\;X}{\Omega }\right)\; & & \;\\ & \;\left(+\frac{k_{3}Y\;X\;\left(X-1\right)}{\Omega^{2}}+k_{4}X\right]\;\mathbb{P}\;\left(X,Y;t\right)\; & & \;\\ & \;+k_{1}\;A\;\mathbb{P}\;\left(X-1,\;Y;t\right)\; & & \;\\ & \;+\frac{k_{2}B\;\left(X+1\right)}{\Omega }\;\mathbb{P}\;\left(X+1,\;Y-1;t\right)\; & & \;\\ & \;+\frac{k_{3}\left(Y+1\right)\;\left(X-1\right)\;\left(X-2\right)}{\Omega^{2}}\; & & \;\\ & \;\mathbb{P}\left(X-1,\;Y+1;t\right)\; & & \;\\ & \;+k_{4}\left(X+1\right)\;\mathbb{P}\;\left(X+1,Y;t\right)\; & & \;\\ & \; & \end{array}$$

Now it is possible to derive the CLE as in (2)

(10) $$\begin{array}{llll} \frac{\text{d}X}{\text{d}t} & =\left[k_{1}A-\frac{k_{2}B\;X}{\Omega }\right)\; & & \;\\ & \left(\;\;+\frac{k_{3}Y\;X\left(X-1\right)}{\Omega^{2}}-k_{4}X\right]\; & & \;\\ & \;\;+\;\sqrt{k_{1}A}\xi_{1}\left(t\right)-\sqrt{\frac{k_{2}\;B\;X}{\Omega }}\xi_{2}\left(t\right)\; & & \;\\ & \;\;+\sqrt{\frac{k_{3}\;Y\;X\left(X-1\right)}{\Omega^{2}}}\xi_{3}\left(t\right)\; & & \;\\ & \;\;\left(\text{\_}\sqrt{k_{4}X}\xi_{4}\left(t\right)\right]\; & & \;\\ \frac{\text{d}Y}{\text{d}t} & =\left[\frac{k_{2}B\;X}{\Omega }\right)\; & & \;\\ & \;\;\left(-\frac{k_{3}Y\;X\left(X-1\right)}{\Omega^{2}}\right]\; & & \;\\ & \;\;+\left[\sqrt{\frac{k_{2}B\;X}{\Omega }}\xi_{2}\left(t\right)\right)\; & & \;\\ & \;\;\left(-\sqrt{\frac{k_{3}Y\;X\left(X-1\right)}{\Omega^{2}}}\xi_{3}\left(t\right)\right]\; & & \;\\ & \; & \end{array}$$

It is easy to extract from (10) the RRE in (3) as

(11) $$\begin{array}{llll} \frac{\text{d}X}{\text{d}t} & =\left[k_{1}A-\frac{k_{2}\;B\;X}{\Omega }\right)\; & & \;\\ & \;\left(+\frac{k_{3}Y\;X\left(X-1\right)}{\Omega^{2}}-k_{4}X\right]\; & & \;\\ \frac{\text{d}Y}{\text{d}t} & =\left[\frac{k_{2}\;B\;X}{\Omega }\right)\; & & \;\\ & \;-\left(\frac{k_{3}\;Y\;X\left(X-1\right)}{\Omega^{2}}\right]\; & & \;\\ & \; & \end{array}$$

Note that in deriving (10) and (11) from (9), the variables *X *and *Y *(which represent molecule numbers, not concentrations, of the species X and Y, respectively) have become continuous instead of remaining discrete. In preparation for phase analysis, some computational quantities have to be derived from (11).

**Figure 8:**
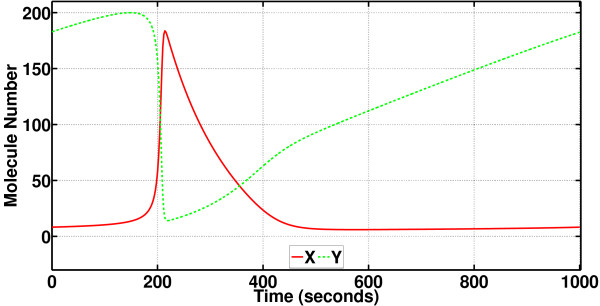
**The periodic solution x**_s_(*t*) **for the Brusselator**. The periodic solution (consisting of the changes in the molecule numbers for the species X and Y) of the RRE (the continuous deterministic model) for the Brusselator. This periodic solution vector function is called **x**_s_(*t*). Note that the oscillating molecular system has discrete states, i.e., it has discrete numbers for the molecule numbers for each species. However, through the continuous-state limit, we have derived the RRE and CLE, which are continuous, from the original oscillator model. Therefore, entries of the periodic solution **x**_s_(*t*) in this figure are continuous valued. Also, in the transformation from the discrete model to the continuous one, we have chosen to stick with molecule numbers for species rather than switching to concentrations, because we would like to plot on top of each other, compare, and use in computational analysis the SSA sample paths obtained from the discrete model and sample paths and deterministic solutions obtained from the continuous models.

The phase analysis of a continuous oscillator (modeled by nonlinear systems of ODEs such as an RRE) depends on linearizations around the steady-state periodic waveform **x**_s_(*t*) solving the RRE. The periodic solution **x**_s_(*t*) for the Brusselator in (6) is given in Figure 8. This function has been computed for a whole period (with the actual approximate value for the period *T *= 1000 s) through the shooting method [40]. The species A, B, R, and S, with their molecule numbers constant, should be excluded from the machinery of the shooting method for it to work.

**Figure 9:**
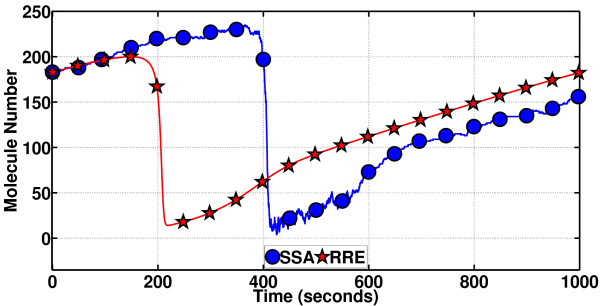
**An SSA-generated sample path as compared to the deterministic periodic solution for the Brusselator (showing changes in the molecule number for only the species Y)**. Changes in the molecule number for only species Y monitored. The noisy sample path is compared to the noiseless RRE solution. The noise has an adverse effect such that it has apparently caused the oscillator to lag behind the deterministic solution. A quantitative measure of this phase shift on a point-by-point basis (for all points) in the sample path is to be obtained by the phase computation methods proposed in this article.

In fact, **x**_s_(*t*) computation is enough preparation for running the brute-force scheme PhCompBF as will be demonstrated next. Recalling that we aim to solve for the possibly constantly changing phase along individual SSA-generated sample paths, we run the SSA algorithm to generate the sample path given in Figure 9. In this plot, the SSA simulation result and the unperturbed **x**_s_(*t*) have been plotted on top of each other, for only species Y, for illustration purposes. It must be noted that both **x**_s_(*t*) and the SSA sample path start initially at the same state on the limit cycle, therefore the star and the circle are on top of each other at *t *= 0 s. Due to isochron-theoretic oscillator phase theory, the initial relative phase, or the initial phase shift of the SSA sample path with respect to **x**_s_(*t*), is zero.

In Figure 9, we would like to solve eventually for the time-evolving relative phase shift of the SSA sample path, for now with PhCompBF. This means solving for the phase shift for the visited states in the sample path, denoted by circles in the figure, and preferably for all the states in between the circles along the path as well. PhCompBF requires running a particular type of simulation for computing the relative phase shift of each visited state. We will demonstrate the method shortly, but let us comment on how much information can be gained by inspecting only the plot in Figure 9. The SSA simulation suggests that the system continually introduces noise, so that everything about the system appears noisy, the phase, the amplitude, etc. Phase is a particular quantity that helps quantify the effect of noise on an autonomously oscillating system. One may easily guess that the relative phase shift of the SSA sample path is always changing along the interval of simulation. It is not obvious at all how to compute this phase shift at particular points in time in Figure 9. Perhaps, one may argue that the sudden decrease that should take place at about *t *= 200 s for the unperturbed **x**_s_(*t*), appears about 200s in time later for the SSA path. However, this is only an educated guess and an approximate value. Also, that the stars and circles appear very close to each other for example in between 600 and 1000s does not directly help invoke the isochron-theoretic phase theory to deduce that the phase shift along this interval is close to zero. Recalling that Figure 9 depicts only species Y, one has to inspect also the other species to arrive at such a conclusion. It is also needless to state as a reminder that for two states to have the same relative phase, having the two states equal to each other is a sufficient but not necessary condition, again due to isochron theory. In all, accurately what happens to the phase shift along the interval is still obscure. As a side note, one should also note that without the perfectly periodic **x**_s_(*t*), it is awfully difficult to guess the period *T*, inspecting only a long SSA sample path. Relevant theory for noisy oscillators suggests that inspecting the zero-crossings of a whole ensemble of long and mildly noisy SSA sample paths yields information related to the period and phase diffusion constant of an oscillator, in a brute-force manner [11].

**Figure 10:**
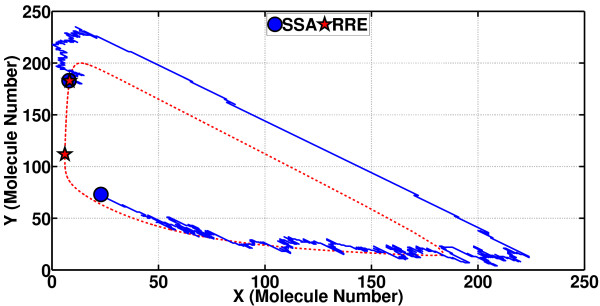
**Limit cycle and SSA sample path shown on the state space for the Brusselator**. Both trajectories start at the same point on the limit cycle. The star traces the limit cycle in clockwise direction, whereas the noisy sample path wanders around though remaining close to the periodic orbit. After some time has passed, the star (of the noiseless path) and the circle (of the noisy sample path) are found to be at different locations. The qualitative difference in terms of phase between these two points is explained by the concept of isochrons.

In order to demostrate PhCompBF, we have first plotted both the SSA sample path and the limit cycle (the closed curve traced over and over by **x**_s_(*t*)) in 2-D state space as in Figure 10. As stated earlier, the star and the circle are initially coincident. Then, as time progresses, **x**_s_(*t*) just traces the limit cycle, but the SSA sample path **x**_ssa_(*t*) runs berserk. At *t*_0 _= 600 s, we have again indicated where the two traces end up. The SSA path at this time is off the limit cycle. Since we do not have exact isochron information, it is not possible to compute the phase $\hat{t}$ value that makes **x**_ssa_(*t*_0 _= 600 s) and $x_{\text{s}}(\hat{t})$ in-phase, i.e., on the same isochron. If we could find this $\hat{t}$ value, then $\alpha (t_{0}=600\text{s})=\hat{t}-600$ would be the sought phase shift value.

**Figure 11:**
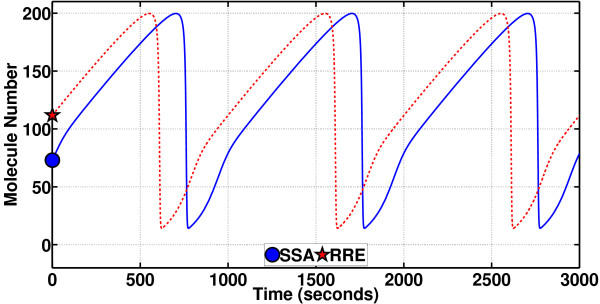
**Mechanism of PhCompBF (the brute-force phase computation scheme) in time domain for the Brusselator (changes in the molecule number for the species Y are monitored)**. The star and the circle obtained at the end of the simulations (let us call this time *t*_0_) in Figure 10 are fed as initial conditions to the RRE in (11), hence the star and the circle at the beginning of the traces in this figure. The waveforms in this figure monitor the same entry for these two different RRE solutions, i.e., the changes in the molecule number for the species Y are shown. The curve starting with the circle should come to be almost periodic in a matter of a few periods for this oscillator. Then the phase shift between the two waveforms can be computed. This phase shift belongs to the point identified by the circle in Figure 10 at the end of the simulation (we have called this time *t*_0_). This phase shift has been obtained with respect to the star in Figure 10 at again the time *t*_0_.

**Figure 12:**
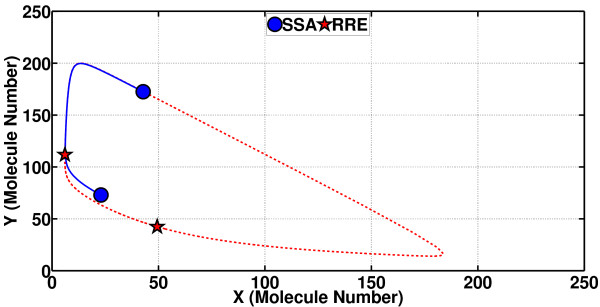
**Mechanism of PhCompBF (the brute-force phase computation scheme) in state space for the Brusselator (changes in molecule numbers for both species are monitored)**. This is the state-space pictorial description of PhCompBF that corresponds to Figure 11. There are two RRE solutions in this figure. The final states of the solutions in Figure 10 are fed as initial conditions to the RRE in (11) for the Brusselator. The RRE solution, whose initial and final conditions are indicated by the circle, approaches the limit cycle and almost starts tracing it, traveling clockwise. The solution indicated by the star, which is actually leading in terms of phase that indicated by the circle, has already made the rightmost turn at the end of this simulation. The circle is way behind. The actual phase shift between the two solutions has to be computed as explained in Figure 11.

The value of the phase shift *α *can, however, be computed through a possibly long, ideally infinitely long, simulation, in line with the theory of asymptotic phase (a theory on intimate terms with isochrons). The following is the essence of PhCompBF. One takes in Figure 10 the states **x**_ssa_(*t*_0 _= 600 s) (the circle on the SSA path) and **x**_s_(*t*_0 _= 600 s) (the star on the limit cycle) and feeds them as initial conditions to the RRE in (21) and then simulates both traces for some time. The result is the two traces in Figure 11. In this plot, again only the species Y is demonstrated. The circular marker (along with the corresponding star) has been put only at the beginning of the simulation in Figure 11 to note the fact that only the initial value belongs to the SSA sample path. After this initial time, both traces are parts of separate RRE solutions. Incorporation of these two new simulated traces into the plot of Figure 10 would be as follows (see Figure 12): The plot starting with the circle in Figure 11 (with both of the two states) would be a curve in the state space of Figure 10 starting from the circle off the limit cycle but gradually converging to it. Meanwhile, the plot starting from the star in Figure 11 would resume tracing the limit cycle in Figure 10 from again the star. Then, as shown in Figure 12, the two simulated plots are observed to be tracing the limit cycle after simulating long enough in time, the star of the unperturbed path always leading the circle of the initially perturbed path (but notice that during the simulation for both traces in Figure 12 all perturbations or noise are removed). Observe in Figure 12 that the star has went ahead to make the rightmost turn on the limit cycle, travelling clockwise, whereas the circle is still way behind. However, all along this simulation of Figure 12, the instantaneous phase shift between the two traces has remained the same. As the simulation goes on along the limit cycle, the circle (originating from the SSA simulation) and the star (of the unperturbed **x**_s_(*t*)) would appear sometimes near, and sometimes far away from each other. This effect is due to particularly the varying velocity along the limit cycle, all determined by the dynamic properties of the RRE. The constant difference in time between the circle and star is the phase shift *α *(*t*_0 _= 600 s) that we aim to compute. Notice that in the state space of Figures 10 and 12, time is only an implicit parameter. Therefore, we have to inspect plots of the type in Figure 11 to obtain the desired phase shift value.

For some oscillators (as determined by the dynamics of the RRE again), a state off the limit cycle converges fast to begin tracing quickly an almost periodic curve, as in the case in hand. Almost two periods are enough to deduce the phase shift between the two curves. After RRE simulations, the phase shift can be computed using Fourier transforms [15].

One question that may arise is why we are particularly using the traces belonging to the species Y to compute phase shifts in Figure 11. Indeed, it follows from the theory that phase is a scalar-valued property of the whole system, therefore investigating phase shifts over non-constant periodic molecule numbers for any species in a system would yield the same phase shift value. In this case, employing Y is only a matter of choice.

Notice that this brute-force scheme is carried out to compute the relative phase shift of the SSA sample path at only *t*_0 _= 600 s. The phase shift for each state along the sample path can be computed one by one through the just outlined PhCompBF.

**Figure 13:**
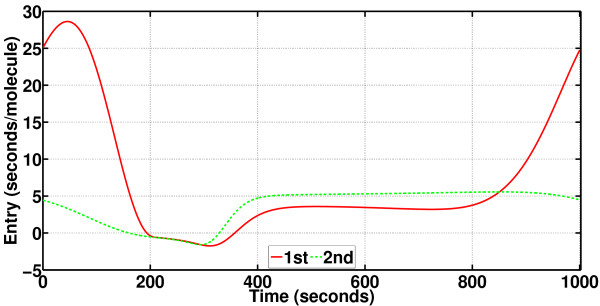
**Phase gradient for the Brusselator**. Entries of the phase gradient (a vector function) as periodic functions, computed through the algorithm described in [11]. The phase gradient is referred to as **v**(*t*) in this article.

**Figure 14:**
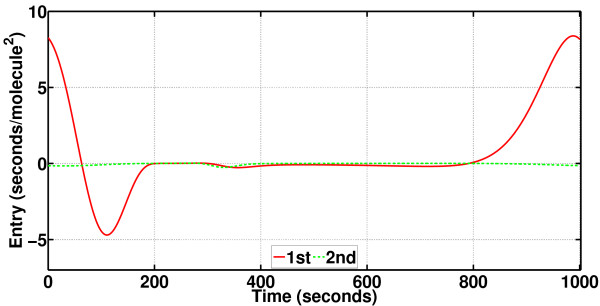
**Phase Hessian diagonals for the Brusselator**. Diagonal entries of the phase Hessian (a square matrix function) as periodic functions, computed through the algorithm described in [15]. The phase Hessian is referred to as **H**(*t*) in this article.

It has already been stated that PhCompBF is almost the golden reference for phase computations but also that the method is very time-consuming. It was for this reason that new methods depending on isochron and orbital deviation approximations were proposed. Particularly, two quantities are necessary for characterizing isochron approximations: the phase gradient **v**(*t*) and the phase Hessian **H**(*t*). These are depicted for the Brusselator respectively in Figures 13 and 14. Recall that **v**(*t*) is a vector function, but **H**(*t*) is a matrix function. Therefore, only the phase Hessian diagonals have been plotted in Figure 14.

Phase computation schemes are fairly easy to comprehend geometrically. Regarding for example the limit cycle depicted in Figure 10, there are both a hyperplane (accounting for the linear isochron approximation) and a quadric surface (for quadratic approximation) associated with each point on the limit cycle. Equations for these characterizations are given in (40) and (41), respectively. A phase computation scheme aims to solve for that point on the limit cycle whose linear or quadratic isochron approximation passes through a given point, for example the stated point denoted by the circle off the limit cycle in Figure 10, **x**_ssa_(*t*_0 _= 600 s). Notice that PhCompBF is also a variant of these phase computation schemes, but in this case not the isochron approximations but the exact isochrons themselves associated with points on the limit cycle are used.

The geometrical interpretations of phase equations, on the other hand, are not easy to visualize. As stated in previous sections, phase equations are differential equations involving orbital deviation in addition to isochron approximations. Phase computation schemes are expected to be more costly but then more accurate with respect to phase equations. Phase equations, as they are differential equations and need to be discretized, suffer from local truncation errors and global errors, whereas this is not the case for the schemes that are in the form of algebraic equations. An approximate phase computation scheme may deviate from the golden reference (PhCompBF result) at times (particularly if the noisy state is too far off the limit cycle), but the scheme (if carefully designed) does not suffer from the accumulation of truncation errors and its phase results are expected to be almost always very close to that of PhCompBF.

**Figure 15:**
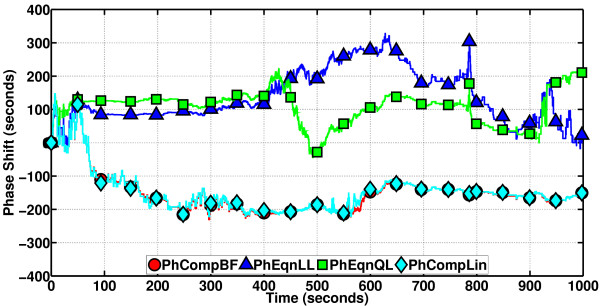
**Phase computation methods on the Brusselator**. The approximate schemes are almost a perfect match for the golden reference PhCompBF. The equations are very fast as indicated by the speed-up figures given in the text. Results of the phase equations are quite close to each other. In the interval 400-600 s, PhEqnQL comes closer to the true value. The following observations and facts are iterated for this first figure of the results. For convenience, these comments are not going to be repeated for every figure that follows. Note that the phase equations are in reality differential equations, solving one by one for the instantaneous phase of points in an oscillator sample path. Therefore, due to the approximations involved in their design and, furthermore, due to the imperfect discretizations (of the differential equations that they are represented by) for their numerical solutions, the phase equations are doomed to suffer from accumulating truncation errors. This is why, in many results figures for the oscillators in this article, we observe the results of phase equations tending to deviate from the golden reference PhCompBF as time progresses. However, computational complexity-wise the phase equations are indeed very fast. This makes the phase equations a feasible and accurate choice for the phase computations of less noisy oscillators, possibly with a dense grid of timepoints in an SSA sample path and high molecule numbers for every species in the system (especially in a container of large volume), deviating not much from their limit cycles. The phase computation schemes, on the other hand, do not employ as many approximations as the phase equations do in their design. Furthermore, these schemes are in the form of algebraic equations, again solving one by one for the instantaneous phase of points in an oscillator sample path. Therefore, the schemes, for their numerical solution, do not involve time discretizations as the phase equations do. This means that the schemes do not suffer from truncation error accumulation. The schemes are subject to errors originating from the approximations committed in their theoretical development, and once again, these approximations are not on the same scale as those employed in the derivation of phase equations, i.e., the schemes are much more accurate than the equations. However, the numerical procedures associated with the schemes render them more costly in computational complexity with respect to the equations. Therefore, one may rightfully contend that the phase computation schemes are tailored to fit phase computations for moderately noisy oscillators in small volume, with low molecule numbers for each species and possibly a sparse grid of timepoints in an SSA sample path.

We now check the performance of the phase computation methods for this oscillator, on a sample path that lasts about 1000 s, with the period about the same as that. The results are depicted in Figure 15. PhCompBF takes about 138 min. Speed-up of the methods on this duration are as follows: PhCompLin (the scheme depending on linear isochron approximations) 56x, PhEqnLL (the phase equation that employs linear isochron approximations and a linear differential equation model for orbital deviations) 8583x, and PhEqnQL (the phase equation with quadratic isochron and linear orbital deviation approximations) 2257x. The phase equations are most of the time sharing a common accuracy level, not disregarding the apparent attempt of PhEqnQL to come closer to PhCompBF around 400-600 s. PhCompLin is slower than the equations but almost as accurate as can be.

### 5.2 Oregonator

In this section, we present phase computation results for a well-known and studied biochemical oscillator, the oregonator [38]. This realistic oscillator accurately models the Belousov-Zhabotinsky reaction, an autocatalytic reaction that serves as a classical example of non-equilibrium thermodynamics. The molecular reactions model, adapted mostly from [39], is given as follows. Names of the reactants have been simplified for convenience.

(12) $$\begin{array}{llll} \text{A}\;\text{ + }\;\text{Y} & \overset{k_{1}}{\to }\;\text{X}\;\text{ + }\;\text{R}\; & & \;\\ \text{X + Y} & \overset{k_{2}}{\to }2\;\text{R}\; & & \;\\ \text{A + X} & \overset{k_{3}}{\to }2\text{X + 2}\;\text{Z}\; & & \;\\ \text{2X} & \overset{k_{4}}{\to }\text{A + R}\; & & \;\\ \text{2}\;\text{B}\;\text{ + }\;\text{2}\;\text{Z} & \overset{k_{5}}{\to }\text{Y}\; & & \;\\ & \; & \end{array}$$

In (12), the propensity functions, employing also the volume of the container, can easily be derived. Parameter values are: *k*_1 _= 0.005 s ^-1 ^mL, *k*_2 _= *k*_3 _= *k*_4 _= 1 s^-1 ^mL, and *k*_5 _= 1.25 *× *10 ^-4 ^s^-1 ^(mL)^3^. Molecule numbers for the reactants A, B, and R are held constant. For this model, the volume initially is set to 12,000 mL. In this case, noise will not have considerable effect on a sample path. Then, we set the volume to 3,200 mL in order to obtain a moderately noisy oscillator. Later on, we will, halve the value of the volume parameter, resulting in a very noisy oscillator, and the performance of the phase computation methods will be demonstrated for this latter case as well.

**Figure 16:**
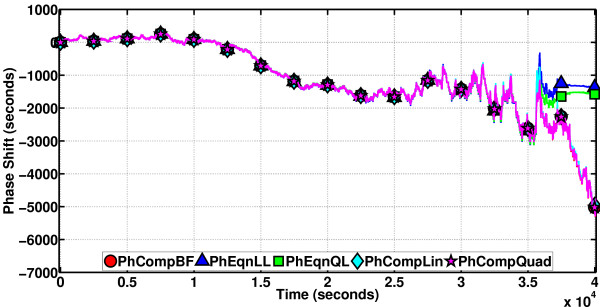
**Phase computation methods on the Oregonator**. (volume = 12, 000 mL). In a large volume, the system is not expected to be very noisy. The phase is closer to ideal. Therefore, all phase computation methods are quite accurate, but the phase equations have started to accumulate global errors towards the end of the simulation, as they are differential equations.

With the volume as 12,000 mL, the performance of the phase computation methods on a particular sample path of length 4 × 10^4 ^s (the period is about 4.43 × 10^4 ^s) is depicted in Figure 16. PhCompBF simulation takes 502 minutes, with two periods of RRE computations before setting out to compute the phase shift values. There are a total of 8114 timepoints on the sample path. As the volume is decreased, the number of timepoints per unit time will reduce. The speed-up of the methods over PhCompBF are: PhCompLin (on linear isochron approximations) 70x, PhEqnLL (on linear isochron and linear orbital deviation approximations) 10733x, PhCompQuad (on quadratic isochron approximations) 46x, and PhEqnQL (on quadratic isochron and linear orbital deviation approximations) 2791x. It is observed that all the methods for a good part of the sample path stick to the PhCompBF result. However, towards the end the phase equations (with PhEqnQL a little more accurate compared to PhEqnLL) begin accumulating global errors, Otherwise, they are exquisitely fast all the time and accurate at the beginning until they start deviating from the golden reference. The phase computation schemes are not as fast as the equations, but they are always accurate in this simulation.

**Figure 17:**
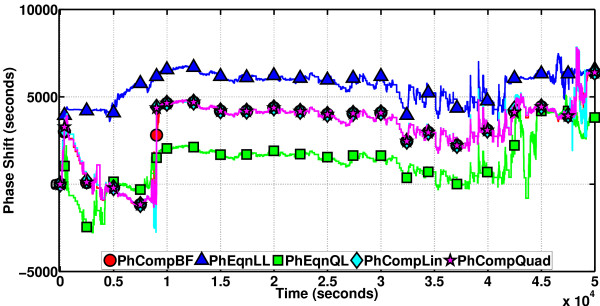
**Phase computation methods on the Oregonator**. (volume = 3, 200 mL). In a smaller volume, the system is noisier. Phase equation results deviate in accuracy. PhEqnQL is a little more accurate. The schemes retain their accuracies.

We have also tested the phase computation methods on a sample path, with the volume set to 3,200 mL. Figure 17 illustrates the results. The simulation interval length (5 × 10^4 ^s) is a little more than the period (about 4.37 × 10^4 ^s). The simulation for PhCompBF took 242 minutes, and there are 2981 timepoints in total. The observed speed-ups were: PhCompLin 70x, PhEqnLL 13971x, PhCompQuad 51x, and PhEqnQL 3203x. It is observed that the phase equations are really fast, keeping track of the exact phase though not very closely, whereas the computation schemes, though not as fast, are almost a perfect match for the exact phase in terms of accuracy.

**Figure 18:**
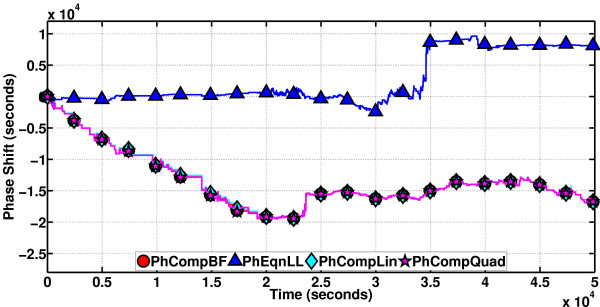
**Phase computation methods on the Oregonator**. (volume = 1, 600 mL). In an even smaller volume, the system is very noisy. PhEqnLL computes the phase shift result very fast, but this result is unacceptably inaccurate. The schemes are still accurate.

We then set volume to 1,600 mL, resulting in a noisier oscillator. We expect the phase equations results to deviate much more from the exact one, and the computation schemes to still do well. Again for a sample path (of length 5 × 10^4 ^s with the period 4.3 × 10^4 ^s), the PhCompBF simulation now takes 76 min. There are 1033 timepoints. Speed-ups with the methods are: 12637x (PhEqnLL), 74x (PhCompLin), and 44x (PhCompQuad). PhEqnQL apparently suffers from numerical problems for such a noisy oscillator, and the result for this method is not included. In Figure 18, we observe in line with our expectations that although PhEqnLL is again very fast, the result it produces is almost unacceptably inaccurate, whereas both the computation schemes maintain their relative speed-ups (as compared to the less noisy version) along with their accuracies.

### 5.3 Repressilator

**Figure 19:**
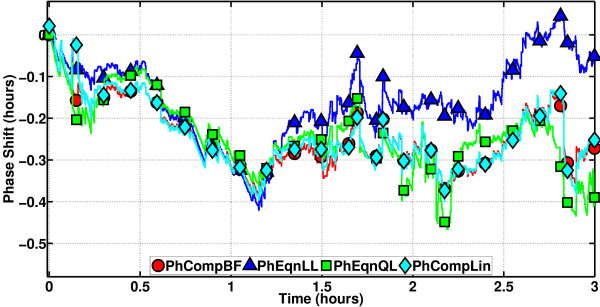
**Phase computation methods on the Repressilator**. PhEqnQL is more accurate than PhEqnLL, but the scheme PhCompLin is the most accurate.

The Repressilator is a synthetic genetic regulatory network, designed from scratch and implemented in *Escherichia coli *using standard molecular biology methods [9]. Its development is a milestone in synthetic biology. We have obtained the model as an SBML file in XML format [41-43]. We have used the libSBML [44] and SBMLToolbox [45] libraries to interpret the model and incorporate it to our own manipulation and simulation toolbox for phase computations. The period of the continuous oscillator obtained from the model is about 2.57 h. A sample path running for about 3 h was generated, and the phase methods were applied. The results are in Figure 19. PhCompBF (the brute-force scheme) takes about 76 min. Speed-ups obtained with the methods are: PhCompLin (on linear isochron approximations) 58x, PhEqnLL (on linear isochron and linear orbital deviation approximations) 7601x, and PhEqnQL (on quadratic isochron and linear orbital deviation approximations) 1994x. It appears in Figure 19 that PhEqnLL towards the end of the simulation has started to accumulate a global error. PhEqnQL looks a little more accurate. Again PhCompLin is, excepting a few minor intervals, the most accurate.

## 6 Conclusions and future work

The phase computation methods described in this article basically target three classes of discrete molecular oscillators. First, the continuous phase models, based on the information obtained from the oscillator model in the continuous-state limit (i.e., basically the limit cycle and isochron approximations), are acceptably accurate for discrete molecular oscillators with a large number of molecules for each species, in a big volume. Indeed, we have shown in this article that the phase equations serve this purpose well. Second, for oscillators with very few molecules for each species in a small volume, a new phase concept needs to be developed, without resorting to continuous limit approximations. This one is as yet an unsolved problem. Third, there are systems in between the two classes just stated, with moderate number of molecules, for which the continuous phase concept is still useful but requires a hybrid approach with combined use of both discrete and continuous models for acceptable accuracy (note that the phase computation schemes are tailored to concretize this hybrid approach), and this is where the contribution of this article should be placed.

As yet, the described methods benefit extensively from continuous state-space approxi-mations derived from the molecular descriptions of such oscillators, and the assumed most accurate brute-force scheme shares this aspect. A future direction furthering this study can be described as follows, in line with the necessity of handling the second class of oscillators stated above. A proper phase model theory (not relying on continuous limit approximations) for discrete-space oscillators modeled with Markov chains needs to be developed. We believe that such a discrete phase model theory can be developed based on *cycle representations *for Markov chains [46-48]. We made progress also on this problem. We have developed a theory that precisely characterizes the phase noise of a single cycle in a continuous-time Markov chain. We were able to show that the phase noise theory we have developed for a single cycle in fact reduces to the previously developed continuous-space phase noise theory in the limit. We are currently working on extending this discrete phase noise theory to many cycles, i.e., to a *cycle decomposition *of a continuous-time Markov chain.

## 7 Methods - Modeling and simulation of discrete molecular oscillators

In this section we review, after giving preliminary information (Section 7.1), some crucial paradigms in the modeling of discrete molecular oscillators: a model that is the complete probabilistic characterization of a discrete system, known as the CME (Section 7.2), a continuous deterministic approximation to the CME in the form of the Reaction Rate Equation (Section 7.3), and the steps that let us proceed to a continuous stochastic model, the Chemical Langevin Equation, from again the CME (Section 7.4). Also a descriptive review of the SSA algorithm of Gillespie [25] for the simulation of molecular models is provided in Section 7.5.

### 7.1 Preliminaries

We first describe a mathematical model for an autonomous, discrete molecular oscillator based on a stochastic chemical kinetics formalism [24-28,30]. We consider *N *molecular species denoted by S_1_, S_2_,..., S_*N*_. Let **X **be the stochastic vector [*X*_1, _*X*_2_, ..., *X*_*N*_]^Τ ^where *X*_*i *_is the number of molecules of species S_*i *_in the reaction chamber (i.e., a cell). The *M *reactions taking place among these molecular species are denoted by R_1_, R_2_, ..., R_*M*_. Let *a*_*j *_(**X**) denote the *propensity *[25,27] of reaction *j*, i.e., the probability that one R_*j *_reaction will occur somewhere in the system in the next infinitesimal time interval [*t*, *t *+ d*t*) is given by *a*_*j *_(**X**) d*t*, i.e.,

(13) $$\mathbb{P}\;\left({\text{R}}_{j}\text{ occurs}\;\text{in}\left[t,t+\text{d}t\right)\right)=a_{j}\left(X\right)\text{d}t$$

Let *s*_*ji *_denote the change in the number of molecules of species S_*i *_as a result of one R_*j *_reaction. We define the stoichiometry vector **s**_*j*_

(14) $$s_{j}={\left[s_{j1},s_{j2},...,s_{jN}\right]}^{⊺}$$

for reaction R_*j*_, and the *N *× *M *stochiometry matrix [27]

(15) $$S=\left[s_{1},s_{2},\ldots ,s_{M}\right]$$

### 7.2 Chemical master equation

The following derivation follows closely that outlined in [27]. Let us take a note of the events **X**(*t *+ d*t*) = **x**, **X**(*t*) = **x **- **s**_*j *_and **X**(*t*) = **x**, where d*t *is an infinitesimal time element. Through several manipulations making use of these events and taking the limit as d*t *→ 0 [27], we obtain

(16) $$\begin{array}{l} \frac{\text{d}\;\mathbb{P}(x,t)}{\text{d}t}=\\ \sum_{j=1}^{M}\left[a_{j}(x-s_{j})\;\mathbb{P}\;(x-s_{j},t)-a_{j}(x)\;\mathbb{P}\;(x,t\right)] \end{array}$$

where ℙ(x,t) denotes the probability that the system is at state **x **at time *t*. The above is known as the CME [27-30]. If we enumerate all the (discrete) state configurations **X **can be in as C_1_, C_2_,..., C_*ns *_and define,

(17) $$p_{i}\left(t\right)=\mathbb{P}\left(x={\text{C}}_{i},t\right)$$

(18) $$p(t)={\left[p_{1}(t),\;p_{2}(t),\;\ldots ,\;p_{ns}(t)\right]}^{\text{T}}$$

then, the CME in (16) can be written as

(19) $$\frac{\text{d}\;p\left(t\right)}{\text{d}t}=Q\;p\left(t\right)$$

where **Q **is a constant square matrix with dimension *ns *× *ns*, known as the *transition rate matrix *[28,29]. The above is a linear system of homogeneous ODEs, but the number of state configurations *ns *is possibly huge. It is usually not practically feasible to construct and solve (19). CME in (16) and (19) above corresponds to a homogeneous, continuous-time Markov chain model [28-30]. The state transitions of this Markov chain are highly structured and compactly described by the list of the reactions as in the CME. The CME provides the ultimate probabilistic characterization for a discrete molecular oscillator. It was shown that the solution of the CME converges to a unique stationary distribution. For a discrete molecular oscillator with a limit cycle, this stationary probability distribution takes the form of a "probability crater" for a planar system with two species [47].

### 7.3 From the stochastic CME to the deterministic rate equations

If we multiply both sides of CME in (16) with **x **and sum over all **x**, we obtain, as shown especially in [24,27],

(20) $$\frac{\text{d}E[X(t)]}{\text{d}t}=\sum_{j=1}^{M}{\text{s}}_{j}\;E[a_{j}(X(t))]$$

We note here that $E\;\left[a_{j}\left(X\left(t\right)\right)\right]\neq a_{j}\left(E\;\left[X\left(t\right)\right]\right)$ unless *a*_*j*_(**x**) is a linear function of **x**. Thus, in general, (20) can not be solved for E[(X(t)] since the term $a_{j}\left(E\;[\left(X\left(t\right)\right]\right)$ involves higher-order moments of **X**(*t*) [27]. However, if we assume that the fluctuations of **X**(*t*) around its mean E[(X(t)] is negligible and thus can perform a crude moment closure scheme, i.e., if E[(X(t)]=X(t), then (20) simplifies to

(21) $$\frac{\text{d}X(t)}{\text{d}t}=\sum_{j=1}^{M}s_{j\;}a_{j}(X(t))=S\;a(X(t))$$

where **S **is the stoichiometry matrix defined in (15) and

(22) $$\text{a}\left(X\left(t\right)\right)={\left[a_{1}\left(X\left(t\right)\right),\;a_{2}\left(X\left(t\right)\right),\;\ldots ,\;a_{M}\left(X\left(t\right)\right)\right]}^{⊺}$$

is an *M *× 1 column vector of reaction propensities evaluated at **X**(*t*). The above system of deterministic ODEs in (21) is known as the RRE [24,27].

### 7.4 From CME to Langevin model

The derivations in this section have been particularly borrowed from [26]. If we assume that the reaction propensities *a*_*j*_(**X**(*t*)) for *j *= 1, ..., *M *are constant in [*t*, *t *+ d*t*) (known as the *leap condition*) [26,27], then the number of the times reactions fire in [*t*, *t *+ *τ*) are independent Poisson random variables [26-30] with mean and variance equal to *a*_*j*_(**x**(*t*)) *τ*, denoted by $P_{j}\left(a_{j}\left(x\left(t\right)\right)\tau \right)$ for *j *= 1, ..., *M*. Hence, we can write,

(23) $$X(t+\tau )=X(t)+\sum_{j=1}^{M}P_{j}(a_{j}(X(t))\tau )s_{j}$$

If we further assume that *a*_*j*_(**x**(*t*)) *τ *≫ 1, then $P_{j}\left(a_{j}\left(x\left(t\right)\right)\tau \right)$ can be approximated with Gaussian random variables:

(24) $$P_{j}\left(a_{j}\left(x\left(t\right)\right)\tau \right)\approx a_{j}\left(x\left(t\right)\right)\tau +\sqrt{a_{j}\left(x\left(t\right)\right)\tau }\;N_{j}\left(0,1\right)$$

where $N_{j}\left(0,1\right)$ for *j *= 1, ..., *M *are independent Gaussian random variables with zero mean and unity variance [26-30]. Incorporating (24) into (23), we recognize the (forward) Euler discretization of the following *stochastic differential equation *(SDE), known as a *Langevin equation *[26-28,30]:

(25) $$\frac{\text{d}\;X\left(t\right)}{\text{d}t}\;=\;S\;a\left(X\left(t\right)\right)\;+\;SD\;\left(\left[\sqrt{a\left(X\left(t\right)\right)}\right]\right)\;\xi \left(t\right)$$

where *ξ*(*t*) denotes an *M *× 1 vector of independent white stationary Gaussian processes with unity (two-sided) spectral density, and

(26) $$\begin{array}{l} D\;\left(\left[\sqrt{a(X(t))}\right]\right)=\\ \left[\begin{array}{ccccc} \sqrt{a_{1}(X(t))} & 0 & \ldots & \ldots & 0\\ 0 & \sqrt{a_{2}(X(t))} & 0 & \ldots & 0\\ \vdots & 0 & \ddots & \ddots & \vdots \\ \vdots & \vdots & \ddots & \ddots & 0\\ 0 & 0 & \ldots & 0 & \sqrt{a_{M}(X(t))} \end{array}\right] \end{array}$$

denotes the diagonal *M *× *M *matrix function shown in (25). We note here that if the stochastic, fluctuation term (known as the *diffusion *term) above is omitted, we obtain the RREs in (21). We note here that, with the Langevin model, the stochastic fluctuations in the oscillator are captured by the second term in the right hand side in (25). This term represents an *additive *noise in the model. By zeroing this additive noise term, we are able to obtain the mean, deterministic dynamics of the oscillator as the solution of the RREs in (21). On the other hand, in the discrete, Markov chain model of the oscillator, the mean, deterministic behavior of the system and the stochastic fluctuations are not separable from each other [26-28,30].

### 7.5 Stochastic simulation algorithm (SSA)

Even though the CME in (16) and (19) provides the ultimate probabilistic characterization for a discrete molecular oscillator, its solution is most often not practical due to the huge number of possible state configurations. As a result, one most often performs a stochastic simulation of the continuous-time Markov chain that models the oscillator and generates a sample path or a realization for the state vector **X**(*t*) as a function of time *t*. This kind of a simulation can be performed with a technique called the SSA, proposed in Gillespie's seminal work [25]. In the original SSA algorithm [25], the computational cost per reaction event (due to the generation of a random variable from a dynamic discrete probability distribution) is O(M) in the number of reactions *M*. The cost per reaction event can be reduced to O(logM) by using a binary tree for random selection of reactions [49], and to $O\left(1\right)$ under certain conditions [50]. One also has to consider the fact that the time gap between reactions tends to shrink as the number of reactions *M*, the number of species *N*, and the number of molecules of every species increases. This means that the total computational cost of SSA for a given time period increases as a result [24]. On the other hand, if the numbers of molecules of all of the species are very large, discrete stochastic simulation of a discrete molecular oscillator in the sense of SSA may be unnecessary [24,27]. In this case, the fluctuations around the deterministic limit cycle will be small, and the continuous Langevin model in (25) may be adequate. As the number of molecules increase, the reaction propensities *a*_*j*_(**X**(*t*)) become larger, and the fluctuation term in the Langevin model in (25) become less and less pronounced in comparison with the drift term, since the magnitude of the drift term is proportional to the reaction propensities whereas the fluctuation term is proportional to their square root [26-28].

Molecular models, their nature (as discrete or continuous, and as stochastic or deterministic), and the algorithms to solve these models are summarized in Figure 1. The approximation that leads us from the discrete stochastic CME to the continuous stochastic CLE is the Gaussian approximation to Poisson random variables and accordingly the *τ*-leap approximation. Similarly, infinite volume approximation takes us from the CLE to the continuous deterministic RRE. Sample paths in line with the CME can be generated through SSA. CLE is a type of stochastic differential equation, so it can be solved via appropriate algorithms. Solution of the RRE requires algorithms designed for ordinary differential equations (ODEs) [26-28].

## 8 Methods - Phase computations based on Langevin models

There exists a well developed theory and numerical techniques for phase characterizations of oscillators with continuous-space models based on differential and stochastic differential equations [15,22]. As described in Sections 7.3 and 7.4, continuous models in the form of differential and stochastic differential equations can be constructed in a straightforward manner for discrete molecular oscillators. Thus, one can in principle apply the previously developed phase models and computation techniques [15,22] to these continuous models.

The outline of this section is as follows: After presenting the preliminaries (Section 8.1), the phase computation problem is introduced (Section 8.2). The methods in Section 8.3 (phase models in the form of ODEs) and in Section 8.4 (phase computation schemes that involve the numerical solution of certain algebraic equations) are designed to numerically solve the phase computation problem of Section 8.2.

### 8.1 Preliminaries

For a molecular oscillator, we assume that the deterministic RREs in (21) have a stable periodic solution **x**_s_(*t*) (with period *T *) that represents a periodic orbit or limit cycle.

An isochron of an oscillator associated with the limit cycle **x**_s_(*t*) is a set of points that have the same phase. For an *N*-dimensional oscillator, each isochron is an *N-*1*-dimensional hypersurface*. The union of isochrons covers the neighborhood of its periodic orbit [1,14]. Isochrons form the basis for phase definition and phase computations for oscillators [22]. In Figure 3, the limit cycle and the isochron portrait of a simple polar oscillator are shown [2,15].

Expanding (21) to first-order (linearization) around **x**_s_(*t*), with

(27) $${\left(G\left(t\right)=G\left(x_{\text{s}}\left(t\right)\right)=\frac{\partial S\;a\left(x\right)}{\partial x}\right|}_{x=x_{\text{s}}\left(t\right)}$$

yields

(28) $$\frac{\text{d}y}{\text{d}t}=G(t)y$$

(28) is a linear periodically time-varying (LPTV) system. The adjoint form of (28) is given by

(29) $$\frac{\text{d}}{\text{d}t}z=-G^{\text{T}}(t)\;z$$

The PPV **v**(*t*) is defined as the *T*-periodic solution of the adjoint LPTV equation in (29), which satisfies the following *normalization condition*

(30) $$v^{\text{T}}(t)\frac{\text{d}\;x_{\text{s}}(t)}{\text{d}t}=u^{\text{T}}(t)\frac{\text{d}\;u(t)}{\text{d}t}=1$$

where **u**(*t*) = d**x**_s_(*t*)/d*t*. The entries of the PPV are the infinitesimal PRCs [1]. The PPV is instrumental in forming linear approximations for the isochrons of an oscillator and in fact is the *gradient *of the *phase *of an oscillator [22] on the limit cycle represented by **x**_s_(*t*).

We next define the matrix **H**(*t*) as the Jacobian of the PPV as follows

(31) $${H(t)=H(x_{\text{s}}(t))=\frac{\partial v(x_{s}(t))}{\partial x_{\text{s}}(t)}=\frac{\partial v(x)}{\partial x}|}_{x=x_{s}(t)}$$

taking into note that actually both **v**(*t*) = **v**(**x**_s_(*t*)) and **H**(*t*) = **H**(**x**_s_(*t*)) are functions of the periodic solution **x**_s_(*t*). The function **H**(*t*) is in fact the *Hessian *of the phase of an oscillator [22] on the limit cycle represented by **x**_s_(*t*). This matrix function is useful in forming quadratic approximations for the isochrons of an oscillator.

### 8.2 Phase computation problem

The phase computation problem for oscillators can be stated as follows. It is observed in Figure 2 that assuming an SSA sample path and the periodic RRE solution start at the same point on the limit cycle (note that the two are in-phase initially), the two trajectories may end up on different isochrons instantaneously at *t *= *t*_0 _(i.e., the two traces at this instant are out of phase). However, according to the properties of isochrons, there is always a point on the limit cycle that is in-phase with a particular point near the limit cycle. Therefore, the existence of $x_{\text{s}}(\hat{t})$ in-phase with the instantaneous point **x**_ssa_(*t*_0_) is guaranteed. We call then the time argument $\hat{t}$ of $x_{\text{s}}(\hat{t})$ the instantaneous *phase *of **x**_ssa_(*t*_0_) [1,2,14,22]. All methods described below in this section are designed to numerically compute this phase value.

### 8.3 Phase equations based on Langevin models

In this section, oscillator phase models in the form of ODEs are described. In [22], we have reviewed the first order phase equation based on linear isochron approximations, and we have also developed novel and more accurate second order phase equations depending on quadratic approximations for isochrons. We will, furthermore in this section, explain how to apply these models to discrete oscillator phase computation.

#### 8.3.1 First-order phase equation based on linear isochron approximations

The first-order phase equation based on linear isochron approximations can be derived from the continuous Langevin model in (25) using the theory and numerical techniques described in [15,22], which takes the form

(32) $$\frac{\text{d}\hat{t}}{\text{d}t}=1+v^{\text{T}}(\hat{t})S\;D\;\left(\left[\sqrt{a(x_{\text{s}}(\hat{t}))}\right]\right)\;\xi (t),\hat{t}(0)=0,$$

where $\hat{t}$ represents the total phase of the oscillator (in units of time) and **v**(*t*) is the PPV discussed above. The value $x_{\text{s}}(\hat{t})$, the periodic solution **x**_s_(*t*) evaluated at the perturbed phase $\hat{t}$, represents possibly a good approximation for the solution of the Langevin equation in (25) provided that the perturbed oscillator does not wander off too far away from the deterministic limit cycle represented by **x**_s_(*t*).

The phase $\hat{t}$ defined above and the phase equation in (32), capture the deviations (from the periodic steady-state) of the perturbed oscillator only along the limit cycle, i.e., phase deviations. A perturbed oscillator also exhibits orbital deviations away from its deterministic limit cycle. Moreover, for a discrete, molecular oscillator, the deterministic periodic solution **x**_s_(*t*) is merely the solution of its continuous and deterministic limit when the number of molecules are assumed to be very large. As such, the solution of a discrete molecular oscillator may exhibit large fluctuations around this continuous and deterministic limit. Thus, $x_{\text{s}}(\hat{t})$ may not serve as a good approximation in such a case. In order to truly assess the quality of $x_{\text{s}}(\hat{t})$ as an approximation in a meaningful manner, we need to compare it with a sample path solution of the discrete, Markov chain model that can be generated with an SSA simulation. However, a one-to-one comparison of $x_{\text{s}}(\hat{t})$ based on the solution of the phase equation in (32) and a sample path obtained with an SSA simulation is not straightforward. In solving (32), one would normally generate sample paths for the independent white stationary Gaussian processes denoted by *ξ*(*t*). In an SSA simulation, sample paths are generated as described in Section 7.5. If done so, a one-to-one comparison between a sample path from an SSA simulation and $x_{\text{s}}(\hat{t})$ would not make sense. In order to make this sample path based comparison meaningful, we use the same discrete random events that are generated in an SSA simulation in order to synthesize the sample paths for the independent white stationary Gaussian processes *ξ*(*t*) in the numerical simulation of (25). More precisely, we proceed as follows. We numerically compute the solution of (32) in parallel and synchronous with an SSA simulation. We discretize the SDE in (32) using time steps that are dictated by the reaction occurrence times in the SSA simulation. Assuming that the last reaction has just occurred at time *t*, the next reaction will occur at time *t *+ *τ *and it will be the *j*th reaction, we form the update equation for $\hat{t}$ as follows

(33) $$\hat{t}(t+\tau )=\hat{t}(t)\;+\;\tau \;+\;v^{\text{T}}(\hat{t}(t))S\;\left[e_{j}-a(x_{\text{s}}(\hat{t}(t)))\tau \right]$$

where **e**_*j *_is the *M *× 1 unit vector with the *j*th entry set to 1 and the rest of the entries set to 0, and

(34) $$a(x_{\text{s}}(\hat{t}))={\left[a_{1}(x_{\text{s}}(\hat{t})),a_{2}(x_{\text{s}}(\hat{t})),\ldots ,a_{M}(x_{\text{s}}(\hat{t}))\right]}^{\text{T}}$$

is an *M *× 1 column vector of reaction propensities evaluated at $x_{\text{s}}(\hat{t})$. The form of the update rule above in (33) can be deduced by examining (24) where we have approximated a Poisson random variable with a Gaussian one. With (33) above, the sample paths for the white Gaussian processes *ξ*(*t*) in (25) (and hence the Wiener processes as their integral) are being generated as a cumulation of the individual events, i.e., reactions, that occur in the SSA simulation of the oscillator at a discrete, molecular level. In the update rule (33), we subtract $a\;(x_{\text{s}}(\hat{t}(t)))\;\tau$ from **e**_*j *_that represents an individual reaction event in order to make the synthesized *ξ*_*j*_(*t*) zero mean. The mean, deterministic behavior of the oscillator is captured by the first drift term on the right hand side of (25) which is used in the computation of the periodic steady-state solution **x**_s_(*t*) and the PPV **v**(*t*). Thus, the mean behavior is already captured, and that is why, it needs to be subtracted in (33). We can now compare $x_{\text{s}}(\hat{t})$ and the SSA generated sample path in a one-to-one manner in order to assess the quality of $x_{\text{s}}(\hat{t})$. We should note here that the SSA simulation that is run in parallel and synchronous with the solution of the phase equation in (32) is necessary only for a meaningful sample path based comparison. One would normally not run an SSA simulation but simply generate sample paths for the Gaussian processes *ξ*(*t*) and numerically solve (32) with an appropriate technique and generate a sample path for the phase $\hat{t}$. In this case, we would not be synthesizing *ξ*(*t*) as a cumulation of reaction events from SSA, but instead directly as white Gaussian processes.

Figure 4 summarizes the phase equations (as opposed to the phase computation schemes, to be introduced later) approach for oscillator phase computations. An SSA sample path is generated. Then, the reaction events in the SSA sample path are recorded. This information, along with limit cycle and isochron approximations computed from the RRE, are fed into phase equations (the first-order phase equation in (32) has been given as an example in Figure 4), which in turn yield the phase $\hat{t}$. A high-level pseudocode description of phase computations using the first order phase equation is given in Algorithm 1.

In (33), we evaluate the reaction propensities at $x_{\text{s}}(\hat{t})$, on the solution of the system projected onto the limit cycle represented by **x**_s_(*t*). However, the oscillator also experiences orbital fluctuations and rarely stays on its limit cycle. Based on linear isochron approximations, we can in fact compute an approximation for the orbital fluctuations as well by solving the following equation [22]

(35) $$\begin{array}{l} \frac{\text{d}\;Y(t)}{\text{d}t}=G(\hat{t})\;Y(t)\;+\;S\;D\;\left(\left[\sqrt{a(x_{\text{s}}(\hat{t}))}\right]\right)\;\xi (t)\\ \;\;\;-\left[v^{\text{T}}(t)\;S\;D\;\left(\left[\sqrt{a(x_{\text{s}}(\hat{t}))}\right]\right)\;\xi (t)\right]\;u(\hat{t}) \end{array}$$

With the orbital fluctuation computed by solving the above linear system of differential equations, we can form a better approximation for the solution of the oscillator:

(36) $$X(t)\approx x_{\text{s}}(\hat{t})+Y(t)$$

Then, one can evaluate the reaction propensities at $x_{\text{s}}(\hat{t})+Y(t)$ instead of $x_{\text{s}}(\hat{t})$, in (32), (33) and (35), in order to improve the accuracy of phase computations. One can further improve accuracy, by replacing $G\;(\hat{t})$ in (35) with

(37) $${G(x_{\text{s}}(\hat{t})+Y(t))=\frac{\partial S\;a(x)}{\partial x}|}_{x=x_{\text{s}}(\hat{t})+Y(t)}$$

marking also that the matrix **G **is indeed a function of explicitly the state variables. Still, the equations in (32) and (35) are both based on linear isochron approximations. Phase and orbital deviation equations based on quadratic approximations for isochrons will provide even better accuracy, which we discuss next.

#### 8.3.2 Second-order phase equation based on quadratic isochron approximations

The second-order phase equation based on quadratic isochron approximations can be derived from the continuous Langevin model in (25) using the theory and numerical techniques described in [15,22], which takes the form

(38) $$\begin{array}{lll} \frac{\text{d}\hat{t}}{\text{d}t}=1+{\left[v\left(\hat{t}\right)+H\left(\hat{t}\right)\;Y\left(t\right)\right]}^{⊺}\;S\;D\;\left(\left[\sqrt{a\left(x\left(t\right)\right)}\right]\right)\;\xi \left(t\right) & \; & \\ \;\hat{t}\left(0\right)=0, & \; & \\ & \; & \end{array}$$

with

(39) $$\begin{array}{c} {\left(\frac{\text{d}Y\left(t\right)}{\text{d}t}=G\left(\hat{t}\right)\;Y\left(t\right)+\frac{1}{2}\frac{\partial G\left(x\right)}{\partial x}\right|}_{x=x_{\text{s}}\left(t\right)}\left(Y\left(t\right)⊗Y\left(t\right)\right)\\ \;\;\;\;+S\;D\;\left(\left[\sqrt{a\left(X\left(t\right)\right)}\right]\right)\;\xi \left(t\right)\\ \;\;\;\;-\left\{{\left[v\left(\hat{t}\right)+H\left(\hat{t}\right)Y\left(t\right)\right]}^{\text{T}}\;S\;D\;\left(\left[\sqrt{a\left(X\left(t\right)\right)}\right]\right)\;\xi \left(t\right)\right\}\\ \;\;\;\;\left[u\left(\hat{t}\right)+G\left(\hat{t}\right)Y\left(t\right)\right] \end{array}$$

where ${\left(X\left(t\right)=x_{\text{s}}\left(\hat{t}\right)+Y\left(t\right),\frac{\partial G\left(x\right)}{\partial x}\right|}_{x=x_{\text{s}}\left(t\right)}$ represents an *N *× *N*^2^, matrix, and ⊗ denotes the, Kronecker product making **Y**(*t*) ⊗ **Y**(*t*) an *N*^2 ^× 1 vector.

With quadratic approximations for the isochrons of the oscillator, the phase computations based on (38) and (39) will be more accurate. We can assess the accuracy of the results obtained with these equations again by numerically solving them in synchronous fashion with an SSA simulation while synthesizing the white Gaussian processes *ξ*(*t*) as a cumulation of the reaction events in SSA, as described in Section 8.3.1.

### 8.4 Phase computation schemes based on Langevin models and SSA simulations

With the phase equations based on linear and quadratic isochron approximations described in Section 8.3, we can compute the phase of an oscillator without having to run SSA simulations based on its discrete, molecular model. We note here again that the SSA simulations described in (32) were necessary only when a one-to-one comparison between the results of phase computations based on phase equations and SSA simulations was required. On the other hand, more accurate phase computations can be attained if they are based on, i.e., use information, from SSA simulations. In this hybrid scheme, we run an SSA simulation based on the discrete, molecular model of the oscillator. For points (in the state-space) on the sample path generated by the SSA simulation, we compute a corresponding phase by essentially determining the isochron on which the point in question lies. Here, one can either employ no approximations for the isochrons or perform phase computations based on linear or quadratic isochron approximations. In [15], we have established the theory for these types of approximate phase computation schemes based on linear and quadratic isochron approximations.

The brute-force phase computations without isochron approximations, which we call Ph-CompBF in short, aims to compute the phase difference between two individual given points, based on the isochron-theoretic phase definition with respect to the periodic solution **x**_s_(*t*) tracing the limit cycle. This method is computationally costly [15,22], as the following explanation based on Figure 5 will reveal. An SSA sample path is computed and the instantanous phase of **x**_ssa_(*t*_0_) is desired to be found. Note that *t*_0 _is a particular value in time. For this purpose, in the transition from Figure 5a to 5b, all noise is switched off and RRE solutions (trajectories in state space) starting from **x**_s_(*t*_0_) (star on the limit cycle) and **x**_ssa_(*t*_0_) (circle off the limit cycle) in Figure 5a are computed. We can compute the phase shift between these two traces only when the off-cycle solution converges as in Figure 5c, that is we will have to integrate RRE for this solution until it becomes approximately periodic in the time domain. In this plot, the illustration has been prepared such that the convergence to the limit cycle takes one period or so, but this may not always be the case. Indeed, ideally this process takes infinite time. This is why the brute-force method is costly. Eventually, the phase shift between the two trajectories can be computed and added to instantaneous time *t*_0_, to compute the phase $\hat{t}$[15,22].

The phase computation based on isochron approximations and SSA simulations proceeds as follows: Let **x**_ssa_(*t*) be the sample path for the state vector of the oscillator that is being computed with SSA. We either solve

(40) $$v^{\text{T}}\left(\hat{t}\right)\left[x_{\text{ssa}}\left(t\right)-x_{\text{s}}\left(\hat{t}\right)\right]=0$$

based on linear isochron approximations or

(41) $$\begin{array}{l} v^{\text{T}}(\hat{t})\left[x_{\text{ssa}}(t)-x_{\text{s}}(\hat{t})\right]+\\ \frac{1}{2}\;{\left[x_{\text{ssa}}(t)-x_{\text{s}}(\hat{t})\right]}^{\text{T}}\;H(\hat{t})\left[x_{\text{ssa}}(t)-x_{\text{s}}(\hat{t})\right]=0 \end{array}$$

based on quadratic isochron approximations for the phase $\hat{t}$ that corresponds to **x**_ssa_(*t*) [15,22]. The above computation needs to be repeated for every time point *t *of interest. Above, for **x**_ssa_(*t*), we essentially determine the isochron (in fact, a linear or quadratic approximation for it) that passes through both the point $x_{\text{s}}(\hat{t})$ on the limit cycle and **x**_ssa_(*t*). The phase of $x_{\text{s}}(\hat{t})$, i.e., $\hat{t}$, is then the phase of **x**_ssa_(*t*) as well since they reside on the same isochron. An illustration of the scheme founded upon linear isochron approximations is given in Figure 6. In this plot, we are looking for an isochron whose linear approximation goes through **x**_ssa_(*t*_0_), and this is the isochron of the point $x_{\text{s}}\left({\hat{t}}_{\text{lin}}\right)$. Notice that the linear approximation (the straight line in Figure 6) is tangent to the isochron of $x_{\text{s}}\left({\hat{t}}_{\text{lin}}\right)$ at exactly $x_{\text{s}}\left({\hat{t}}_{\text{lin}}\right)$. The value ${\hat{t}}_{\text{lin}}$ then is the phase computed by this scheme. Notice that there is some difference between the exact solution $\hat{t}$ and the approximate ${\hat{t}}_{\text{lin}}$. This difference is certain to shrink if the isochrones are locally closer to being linear. For more accurate but still approximate solutions, the quadratic scheme can be used [15,22].

We should note here that, even though **x**_ssa_(*t*) above is computed with an SSA simulation based on the discrete model of the oscillator, the steady-state periodic solution $x_{\text{s}}(\hat{t})$, the phase gradient $v\left(\hat{t}\right)$ and the Hessian $H\left(\hat{t}\right)$ (i.e., all of the information that is used in constructing the isochron approximations) are computed based on the continuous, RRE model of the oscillator [15,22]. The phase computation schemes we describe here can be regarded as *hybrid *techniques that are based both on the continuous, RRE and the discrete, molecular model of the oscillator. On the other hand, the phase computation schemes discussed in Section 8.3 based on phase equations are completely based on the continuous, RRE and Langevin models of the oscillator. Figure 7 explains the ingredients that the phase computation schemes utilize. An SSA sample path is generated (note that alternatively a sample path may be generated through the CLE). From the RRE model, limit cycle information (**x**_s_(*t*)) and isochron approximations (**v**(*t*) and **H**(*t*)) are computed. All this information is fed into the phase computation schemes (in Figure 7 we have given the expression for the scheme utilizing linear approximations for convenience, as this is the method likely to be preferred due to its lower complexity despite its inferior accuracy as compared to the quadratic scheme) and then finally the phase $\hat{t}$ is found. A high level pseudocode of phase computations using the scheme depending on linear isochron approximations is given in Algorithm 2.

## 9 Methods - Oscillator models, numerical methods, and implementation notes

This section briefly describes where suitable oscillator models can be found particularly on the internet and how these models can be modified when possible (Section 9.1), how the obtained ODE models can be handled computationally (Section 9.2), a description of the numerical methods used in the simulations (Section 9.3), and the computational costs that they incur (Section 9.4).

### 9.1 Biochemical oscillator models

Oscillator models for analysis can be found from multiple resources on the web. Models generally come in two separate forms, described briefly as follows.

Models of the first type are translated directly from actual biochemical reactions. Propensities of the reactions are functions of a reaction rate parameter and appropriate algebraic expressions of molecule numbers associated with the reacting species. As such, the propensities are always positive. Moreover, the volume parameter (associated with the container or the cell accommodating the species) can easily be incorporated into the propensity functions. Volume of the cell implies the level of noisiness in the sample path simulations, i.e., basically, the more voluminous a cell, the more the number of each reacting species, and then the closer the sample path solution to the ensemble average. Therefore, one may rightfully declare that every different value for the volume parameter defines a new oscillator to be analyzed, although the mechanism of the reactions and the pattern for the propensities remain the same for a pre-determined setting.

Models of the second type are provided directly as ODE models. In some cases, the propensity functions are difficult to handle, and it is not obvious how the crucial volume parameter can be incorporated into the equations. Then, it happens that analysis of these oscillators is a little restricted, not having the capability to adjust the level of noisiness in a correct and reliable manner. However, in all, the simulations can be carried out for the value of the volume implied by the ODE model.

As to where oscillator models can be found on the web, there are multiple alternatives. http://www.xmds.org/[39] is the website for a simulator, in which particularly models from [38] have been modified in appropriate form to be analyzed. We have benefitted extensively from the models we have obtained from these references, as most of them are models of the first type described above. One of the other alternatives is obtaining ODE models (models of the second type stated above) from online repositories such as [41-43] and manipulate them via appropriate software toolboxes [44,45].

### 9.2 Information computed from the ODE model and SSA

Oscillator models are approximated by ODEs in the deterministic sense, through procedures already explained in the previous sections. Our purpose before handling a sample path generated by SSA is to have available in hand some crucial computational quantities that will help compute the phase along the sample path. All these crucial quantities will be computed using the ODE model. A shooting type of formulation [40] is preferred to obtain the periodic solution, more particularly a number of discrete timepoints for **x**_s_(*t*) along a single period. The shooting method solves this boundary value problem efficiently even for large systems of ODEs [40]. A further key benefit is that by-products of the shooting method can be utilized in solving for **v**(*t*), namely the PPV or the phase gradient [11]. On top of **x**_s_(*t*) and **v**(*t*) and using again the by-products of these computations, **H**(*t*), the phase Hessian, can be obtained through the algorithm proposed in [15]. Now, SSA simulations for the sample paths of the noisy molecular oscillator can be performed [25], and these sample paths are analyzed in terms of phase with the following numerical methods. It should be recalled, however, that during the SSA simulation, also pieces of information have to be stored at each reaction event, conveying which reaction was chosen randomly to be simulated and what were the propensity function values at that particular instant.

### 9.3 Phase simulations

In this section, we provide details concerning the numerical aspects of the proposed phase computation methods.

The brute-force scheme (PhCompBF) (described in Section 8.4) is basically run for all of the timepoints in an SSA-generated sample path, and it is very costly in terms of computation. If **x**_ssa_(*t*_0_) is a timepoint in the sample path (naturally at where a state change takes place) the RRE is integrated with this initial condition at *t *= 0 for a long time so that this deterministic solution settles to the limit cycle in continuous time. The solution of the RRE with the initial condition **x**_s_(*t*_0_) at t=0 can be readily computed, this is a shifted version of the periodic solution **x**_s_(*t*) that is available. If the phase shift between the two solutions is computed, this shift is the phase shift of the sample path **x**_ssa _at *t *= *t*_0 _[15]. Since one generally does not know the phase value at the very first timepoint of an SSA sample path, the brute-force scheme is mandatory in computing this phase value and providing the initial condition, on which all of the other approximate phase computation schemes and equations can operate.

The approximate phase computation schemes [15] (again described in Section 8.4) consist of solving the algebraic equation in (40) or (41), depending on whether linear or quadratic approximations are respectively preferred to be used, and they are also run for all points in the SSA sample path (see Algorithm 2 for the pseudocode of phase computations utilizing the scheme founded upon the linear isochron approximations). Benefitting from the scalar nature of these equations, the bisection method is used extensively in their numerical solution. Details and subtleties involved with these schemes (of considerably less computational load compared to PhCompBF) are provided in [15].

Phase equations [22], described in Section 8.3 are in this context stochastic differential equations, operating on the recorded reaction events of an SSA sample path. The specific discretization scheme applied to the first order phase equation is explained in detail in Section 8.3.1 (see Algorithm 1 for the pseudocode of phase computations with this first order equation). This discretization scheme can be easily extended to the second order phase equation of Section 8.3.2.

We will denote each method analyzed and used in generating results by some abbreviations, for ease of reference. The brute-force scheme explained above is denoted by Ph-CompBF, the scheme depending on linear isochron approximations (summarized by (40)) by PhCompLin, and that depending on quadratic in (41) by PhCompQuad. The first order phase equation of (32) is denoted by PhEqnLL (the first L for linear isochron approximations and the second L for linear orbital deviation approximations). The second order phase equation of (38) and (39) is denoted by PhEqnQQ (Q for both type of approximations, isochron and orbital deviation). We prefer to use instead of PhEqnQQ a simpler, but numerically more reliable, version of the second order equation. This simpler version is described by the equations (38) and (35). Equation (35) is the orbital deviation equation belonging to the first order phase equation theory. In turn, we denote this simpler model by PhEqnQL [22].

### 9.4 Analysis of computational complexities

In this section, we analyze the computational costs of phase computation schemes and phase equations. Let us denote by *N *the number of states in an oscillator, *M *the number of reactions, *K *the number of timepoints along a single period, *L *the number of total timepoints along the interval where a phase computation method is run.

Preliminary statements on computational complexities are as follows. We assume as well-known complexities that **x**_s_(*t*), **G**(*t*) (assumed to be sparse), **u**(*t*) and **v**(*t*) are computable along a single period in O(NK) time. The computation of **H**(*t*) (which is usually not sparse) upon the stated quantities takes $O\left(N^{3}\;K\right)$ time [15]. We assume that if a matrix is sparse, then matrix vector multiplications and solving a linear system of equations involving this matrix can be done in linear time.

For PhCompBF (see Section 8.4 and Figure 5 for explanations), in order to compute the phase of a point **x**_ssa_(*t*_0_), we have to integrate the RRE with initial condition **x**_ssa_(*t*_0_) for an ideally infinite number, namely *n*_per_, of periods, so that the states vector can be assumed more or less to be tracing the limit cycle. If FFT (fast Fourier transform) properties are used to compute the phase shift between periodic waveforms, the overall complexity of PhCompBF can be shown to amount to $O\left(n_{\text{per}}K\;N\;L+L\;K{\text{log}}_{2}K\right)$[22].

The approximate phase computation schemes consist of solving the algebraic equations in (40) or (41) (depending on whether the linear or quadratic scheme is preferred). The bisections method is used to solve these equations. In order to compute the phase value of a particular timepoint, an interval has to be formed. In forming such an interval, we start with an interval, of length *d*_min _and centered around the phase value of the previous timepoint, and double this length value until the interval is certain to contain the phase solution. The allowed maximum interval length is denoted by *d*_max_. Then, the bisections scheme starts to chop down the interval until a tolerance value *d*_tol _for the interval length is reached. See Algorithm 2 for the pseudocode of phase computations using PhCompLin (the scheme depending on linear isochron approximations), based on this explanation. More explanations on the flow of PhCompLin are given in Section 8.4 and Figure 6. The PhCompLin computational complexity can be shown to be

(42) $$O\left(N\;L{\text{log}}_{2}\left\lceil \frac{d_{\text{max}}^{2}}{d_{\text{to}1}d_{\text{min}}}\right\rceil \right)$$

and PhCompQuad (which depends on quadratic isochron approximations) complexity is

(43) $$O\left(N^{2}\;L\;{\text{log}}_{2}\left\lceil \frac{d_{\text{max}}^{2}}{d_{\text{tol}}d_{\text{min}}}\right\rceil \right)$$

based on the explanations above.

The computational complexity expressions for all of the phase computation schemes are summarized in Table 1.

**Table 1:** Computational complexities for the phase computation schemes

Scheme	Computational complexity
PhCompBF	$O\left(n_{\text{per}}K\;N\;L+L\;K{\text{log}}_{2}K\right)$
PhCompLin	$O\left(N\;L{\text{log}}_{2}\left\lceil \frac{d_{\text{max}}^{2}}{d_{\text{to}1}d_{\text{min}}}\right\rceil \right)$
PhCompQuad	$O\left(N^{2}\;L\;{\text{log}}_{2}\left\lceil \frac{d_{\text{max}}^{2}}{d_{\text{tol}}d_{\text{min}}}\right\rceil \right)$

In order of increasing computational complexity, the schemes are PhCompLin (on linear isochron approximations), PhCompQuad (on quadratic isochron approximations), and PhCompBF (with no approximations). We denote by *N *the number of states in an oscillator, *K *the number of timepoints along a single period, and *L *the number of total timepoints along the interval where a phase computation method is run. We have *n*_per _as the number of periods that we simulate the RRE with the initial condition that is off the orbit, so that this solution of the RRE can be expected in practice to settle into periodicity, and the phase value associated with the stated initial condition can be computed (note that this is the essence of PhCompBF). The values *d*_max _and *d*_min _are respectively the maximum and minimum lengths of the interval in which a solution for phase is sought. The value *d*_tol _denotes a tolerance.

Phase equation solution complexities depend (in extreme conditions) mainly on the stoichiometric matrix **S **being sparse (few nonzero entries per row) or totally dense. Note that in realistic problems **S **is observed to be usually sparse. These stated respective conditions lead us to come up with best and worst case complexities. As such, PhEqnLL (the equation employing linear isochron and linear orbital deviation approximations) complexity in the best and worse case can be shown to be O(ML+NL) and O(NML), respectively. PhEqnQL (with quadratic isochron and linear orbital deviation approximations) complexities are $O\left(N^{2}\;L+\;M\;L\right)$ (best case) and $O\left(N^{2}\;L+\;N\;M\;L\right)$ (worst case). Complexities for the phase equations are summarized in Table 2. For a pseudocode of phase computations using PhEqnLL, see the explanation in Section 8.3.1 and Algorithm 1 based on this account.

**Table 2:** Computational complexities for the phase equations

Equation	Complexity (best)	Complexity (worst)
PhEqnLL	O(ML+NL)	O(NML)
PhEqnQL	$O\left(N^{2}\;L+\;M\;L\right)$	$O\left(N^{2}\;L+\;N\;M\;L\right)$

We provide the best and worst case computational complexities for the phase equations, PhEqnLL (on linear isochron and linear orbital deviation approximations) and PhEqnQL (on quadratic isochron and linear orbital deviation approximations), according as the stoichiometric matrix **S **is sparse (few entries per row) or fully dense. Note that the computational load the equations entail are much less than those of the phase computation schemes. We denote by *N *the number of states in an oscillator, *M *the number of possible reactions that can occur, *K *the number of timepoints along a single period, and *L *the number of total timepoints along the interval where a phase computation method is run.

The essence of the above analyses is that there is a trade-off between accuracy and computational complexity [22]. For mildly noisy oscillators, the phase equations should remain somewhat close to the results of the golden reference PhCompBF and the other approximate phase computation schemes, which imitate PhCompBF very successfully with much less computation times. For more noisy oscillators, we should expect the phase computation schemes to do still well, although the phase equations will compute some inaccurate results very fast. PhCompBF is always very slow [22].

## Abbreviations

SSA: Stochastic Simulation Algorithm; CME: Chemical Master Equation; CLE: Chemical Langevin Equation; RRE: Reaction Rate Equation; SDE: Stochastic Differential Equation; ODE: Ordinary Differential Equation; PhCompBF: brute-force phase computation scheme; PhCompLin: phase computation scheme depending on linear approximations for isochrons; PhCompQuad: phase computation scheme depending on quadratic approximations for isochrons; PhEqnLL: phase equation depending on linear approximations for isochrons and linear approximations for orbital deviation; PhEqnQL: phase equation depending on quadratic approximations for isochrons and linear approximations for orbital deviation; PhEqnQQ: phase equation depending on quadratic approximations for isochrons and quadratic approximations for orbital deviation.

## Competing interests

The authors declare that they have no competing interests.

## Algorithm 1 - PhEqnLL pseudocode

**input **: oscModel and ssaPath

**output**: phase and phaseShift of points in ssaPath

//compute limit cycle [40]

**1 x**_s_(*t*) = computeLimitCycle (oscModel);

//compute linear isochron approximations along a single period [11]

**2 v**(*t*) = computePhaseGradient (oscModel);

//obtain SSA path data

**3 **pts = pts in ssaPath;

//compute phase

**4 for ***i *← *1 ***to **size(pts in ssaPath) **do**

   //for the first timepoint, use the brute-force scheme PhCompBF

   //refer to Section 8.4 and Figure 5 for explanations

   //refer to Section 9.4 for computational complexity

**5** **if ***i is equal to 1 ***then**

      //tValue of pts(*i*) : the time at which pts(*i*) occurs

      //value of pts(*i*) : state vector for the oscillator at tValue of pts(*i*)

**6**       phaseShift(*i *) = PhCompBF(oscModel, **x**_s_(*t*), tValue *of* pts(*i*), value *of* pts(*i *));

**7**       phase(*i *) = [ tValue of pts(*i*) ] + phaseShift(*i*);

**8**  end

   //for the other timepoints, use the first order phase equation

   //PhEqnLL update rule is given in (33) of Section 8.3.1

   //more implementation details and computational complexity in Section 9.4

   //stoichiometric matrix (**S**) and propensity function (a(**X**))

   //information are embedded in oscModel

**9** **if ***i is not equal to 1 ***then**

**10**    tau = [ tValue of pts(*i *) ] - [ tValue of pts(*i-1 *) ];

         //Now apply the update rule in (33)

         //reactionNo of pts(*i *) : number of the reaction occuring at tValue of pts(*i *)

         //**e**_*j *_is an *M*-sized vector with its *j *th entry one

**11**    phase(*i*) =

         phase(*i-1 *) + tau + **v**^Τ^(phase(*i-1*)) **S **[**e**_reactionNo __of pts(*i*) _- **a**(**x**_s_(phase(*i-1*))) tau];

**12**    phaseShift (*i*)=phase(*i*)-[tValue of pts(*i*)];

13   end

14 end

## Algorithm 1: PhEqnLL pseudocode

**Extended caption for Algorithm 1**: Lines 1-2 compute the limit cycle and the phase gradient. In lines 4-14, the phase computation is described. Lines 5-8 describe the use of the brute-force scheme PhCompBF for the phase computation of the first timepoint. In lines 9- 13, the phase computation of the other timepoints is accomplished via PhEqnLL (the phase equation founded upon the linear isochron and the linear orbital deviation approximations).

Algorithm 2 - PhCompLin pseudocode

**input **: oscModel and ssaPath

**output**: phase and phaseShift of points in ssaPath

//compute limit cycle [40] and linear isochron approximations [11]

1 **x**_s_(*t*) = computeLimitCycle (oscModel); **v**(*t*) = computePhaseGradient (oscModel);

   //obtain SSA path data

**2 **pts = pts in ssaPath;

   //compute phase

**3 for ***i *← *1 ***to **size(pts in ssaPath) **do**

         //for the first timepoint, use the brute-force scheme PhCompBF

**4**       **if ***i is equal to 1 ***then**

**5**          phaseShift(*i*) = PhCompBF(oscModel, **x**_s_(*t*), tValue *of *pts(*i*), value *of *pts(*i*));

**6**          phase(*i *) = [ tValue of pts(*i*)] + phaseShift(*i *);

**7**        end

        //for the other timepoints, use PhCompLin, see (40) of Section 8.4

        //pictorial description in Figure 6

        //algorithm description and computational complexity in Section 9.4

**8**     **if ***i is not equal to 1 ***then**

          //phase(*i-1 *) used as the midpoint of the interval

**9**       *d *= *d*_min_/2; interval = [ phase(*i-1 *) *- d, *phase(*i-1*) + *d*];

**10**      **while **length(interval) *< d*_max_/2 **do**

               //Check if the solution $\hat{t}$ to the following equality (40) is in interval

               //$v^{⊺}(\hat{t})\left[\left[\text{value of pts(}i\text{)]}-x_{\text{s}}(\hat{t}\right)\right]=0$

**11**         **if ***solution is in *interval **then **break;

**12**         *d *= 2* *d*; interval = [ phase(*i-1*) - *d*, phase(*i-1 *) + *d*];

13       end

            //use bisection method to compute the solution to (40)

**14**       phase(*i *) = BisectionMethod(oscModel, **x**_s_(*t*), **v**(*t*), interval, value *of *pts(*i*));

**15**       phaseShift(*i*) = phase(*i*) - [tValue of pts(*i*)];

16    end

17 end

## Algorithm 2: PhCompLin pseudocode

**Extended caption for Algorithm 2**: Line 1 computes the limit cycle and the phase gradient. In lines 3-17, the phase computation is described. Lines 4-7 describe the use of the brute-force scheme PhCompBF for the phase computation of the first timepoint. In lines 8-16, the phase computation of the other timepoints is accomplished via PhCompLin (the phase computation scheme founded upon the linear approximations for isochrons).

## References

[B1] Izhikevich EM Dynamical Systems in Neuroscience: The Geometry of Excitability and Bursting 2007 MIT Press, Cambridge

[B2] Winfree AT The Geometry of Biological Time 2001 Springer, New York

[B3] Goldbeter A Biochemical Oscillations and Cellular Rythms 1996 Cambridge University Press, Cambridge

[B4] Fu L Lee CC The circadian clock: pacemaker and tumour suppressor Nat Rev Cancer 2003 3 350 361 10.1038/nrc1072 12724733

[B5] Fu L Pelicano H Liu J Huang P Lee C The circadian gene Period2 plays an important role in tumor suppression and DNA damage response in vivo Cell 2002 111 41 50 10.1016/S0092-8674(02)00961-3 12372299

[B6] Davis S Mirick DK Circadian disruption, shift work and the risk of cancer: a summary of the evidence and studies in Seattle Cancer Causes Control 2006 17 539 545 10.1007/s10552-005-9010-9 16596308

[B7] Schernhammer ES Laden F Speizer FE Willett WC Hunter DJ Kawachi I Fuchs CS Colditz GA Night-shift work and risk of colorectal cancer in the nurses' health study J Natl Cancer Inst 2003 95 825 828 10.1093/jnci/95.11.825 12783938

[B8] Straif K Baan R Grosse Y Secretan BE Ghissassi FE Bouvard V Altieri A Benbrahim-Tallaa L Cogliano V Carcinogenicity of shift-work, painting, and fire-fighting Lancet Oncol 2007 12 8 1065 1066 19271347 10.1016/S1470-2045(07)70373-X

[B9] Elowitz MB Leibler S A synthetic oscillatory network of transcriptional regulators Nature 2000 403 6767 335 338 10.1038/35002125 10659856

[B10] Demir A Sangiovanni-Vincentelli A Analysis and Simulation of Noise in Nonlinear Electronic Circuits and Systems 1998 Kluwer Academic Publishers, Boston

[B11] Demir A Mehrotra A Roychowdhury J Phase noise in oscillators: A unifying theory and numerical methods for characterisation IEEE Trans Circ Syst I Fund Theory Appl 2000 47 5 655 674 10.1109/81.847872

[B12] Vilar JMG Kueh HY Barkai N Leibler S Mechanisms of noise-resistance in genetic oscillators Proc Natl Acad Sci USA 2002 99 9 5988 5992 10.1073/pnas.092133899 11972055 PMC122889

[B13] Goldbeter A Computational approaches to cellular rhythms Nature 2002 420 6912 238 245 10.1038/nature01259 12432409

[B14] Josic K Shea-Brown ET Moehlis J Isochron Scholarpedia 2006 1 8 1361 10.4249/scholarpedia.1361

[B15] Suvak O Demir A Quadratic approximations for the isochrons of oscillators: a general theory, advanced numerical methods and accurate phase computations IEEE Trans Comput Aided Design Integr Circ Syst 2010 29 8 1215 1228

[B16] Farkas M Periodic Motions 1994 Springer-Verlag, New York

[B17] Demir A Fully nonlinear oscillator noise analysis: an oscillator with no asymptotic phase Int J Circ Theory and Appl 2007 35 175 203 10.1002/cta.387

[B18] Malkin IG Methods of Poincare and Liapunov in Theory Of Non-Linear Oscillations 1949 Gostexizdat, Moscow

[B19] Kuramato Y Chemical Oscillations, Waves, and Turbulence 1984 Springer-Verlag, New York

[B20] Brown E Moehlis J Holmes P On the phase reduction and response dynamics of neural oscillator populations Neural Comput 2004 16 4 673 715 10.1162/089976604322860668 15025826

[B21] Kaertner FX Analysis of white and *f *^-*α *^noise in oscillators Int J Circ Theory Appl 1990 18 485 519 10.1002/cta.4490180505

[B22] Suvak O Demir A On phase models for oscillators IEEE Trans Comput Aided Design Integr Circ Syst 2011 30 7 972 985

[B23] Suvak O Demir A Phase models and computations for molecular oscillators Proc 8th Internat. Workshop on Computational Systems Biology (WCSB 2011) 2011 ETH Zurich, Switzerland 173 176

[B24] Gillespie DT Stochastic simulation of chemical kinetics Ann Rev Phys Chem 2007 58 35 55 10.1146/annurev.physchem.58.032806.104637 17037977

[B25] Gillespie DT Exact stochastic simulation of coupled chemical reactions J Phys Chem 1977 81 25 2340 2361 10.1021/j100540a008

[B26] Gillespie DT The chemical Langevin equation J Chem Phys 2000 113 1 297 306 10.1063/1.481811

[B27] Higham DJ Modeling and simulating chemical reactions SIAM Rev 2008 50 2 347 368 10.1137/060666457

[B28] van Kampen NG Stochastic Processes in Physics and Chemistry 1992 North-Holland, Amsterdam

[B29] Gardiner CW Handbook of Stochastic Methods for Physics, Chemistry and the Natural Sciences 1983 Springer-Verlag, Berlin

[B30] Wilkinson DJ Stochastic Modelling for System Biology 2006 1 CRC Press, New York

[B31] Amdaoud M Vallade M Weiss-Schaber C Mihalcescu I Cyanobacterial clock, a stable phase oscillator with negligible intercellular coupling Proc Natl Acad Sci USA 2007 104 7051 7056 10.1073/pnas.0609315104 17438272 PMC1855407

[B32] Morelli LG Julicher F Precision of genetic oscillators and clocks Phys Rev Lett 2007 98 22 228101 17677881 10.1103/PhysRevLett.98.228101

[B33] Vance W Ross J Fluctuations near limit cycles in chemical reaction systems J Chem Phys 1996 105 479 487 10.1063/1.471901

[B34] Gaspard P The correlation time of mesoscopic chemical clocks J Chem Phys 2002 117 8905 8916 10.1063/1.1513461

[B35] Koeppl H Hafner M Ganguly A Mehrotra A Deterministic characterization of phase noise in biomolecular oscillators Phys Biol 2011 8 5 055008 10.1088/1478-3975/8/5/055008 21832803

[B36] Tomita T Ohta T Tomita H Irreversible circulation and orbital revolution Prog Theory Phys 1974 52 1744 1765 10.1143/PTP.52.1744

[B37] Gabor D Theory of communication J IEE Lond 1946 93 429 457

[B38] Cropper WH Mathematica Computer Programs for Physical Chemistry 1998 Springer-Verlag, New York

[B39] eXtensible Multi-Dimensional Simulator http://www.xmds.org/

[B40] Kundert K White JK Sangiovanni-Vincentelli A Steady-State Methods for Simulating Analog and Microwave Circuits 1990 Kluwer, Norwell

[B41] Cellerator Model Repository http://www.cellerator.info/nb.html

[B42] Cellular Models http://www.cds.caltech.edu/hsauro/models.htm

[B43] Website E-Cell http://www.e-cell.org/ecell/

[B44] Bornstein BJ Keating SM Jouraku A Hucka M LibSBML: an API library for SBML Bioinformatics 2008 24 6 880 881 10.1093/bioinformatics/btn051 18252737 PMC2517632

[B45] Keating SM Bornstein BJ Finney A Hucka M SBMLToolbox: an SBML toolbox for MAT-LAB users Bioinformatics 2006 22 10 1275 1277 10.1093/bioinformatics/btl111 16574696

[B46] Kalpazidou SL Cycle Representations of Markov Processes 2006 Springer-Verlag, Berlin

[B47] Feistel R Ebeling W Deterministic and stochastic theory of sustained oscillations in autocatalytic reaction systems Phys A Stat Theor Phys 1978 93 1-2 114 137 10.1016/0378-4371(78)90213-3

[B48] Hill TL Free Energy Transduction and Biochemical Cycle Kinetics 1989 Springer-Verlag, New York

[B49] Gibson MA Bruck J Exact stochastic simulation of chemical systems with many species and many channels J Phys Chem A 2000 104 1876 1889 10.1021/jp993732q

[B50] Slepoy A Thompson AP Plimpton SJ A constant-time kinetic Monte Carlo algorithm for simulation of large biochemical reaction networks J Chem Phys 2008 128 205101 10.1063/1.2919546 18513044

